# Comprehensive Analysis of Phylogenetic Relationship and Optimal Codons in Mitochondrial Genomes of the Genus *Pseudogastromyzon*

**DOI:** 10.3390/ani14030495

**Published:** 2024-02-02

**Authors:** Cheng Zhang, Shun Zhang, Zhe Tian, Yajun Wang, Shanliang Xu, Danli Wang

**Affiliations:** 1 School of Marine Science, Ningbo University, Ningbo 315211, China; chengzhangcrab@163.com (C.Z.);; 2 National Engineering Research Laboratory of Marine Biotechnology and Engineering, Ningbo University, Ningbo 315211, China

**Keywords:** *Pseudogastromyzon*, mitochondrial genome, phylogenetic relationship, evolution, optimal codons

## Abstract

**Simple Summary:**

The phylogenetic status and evolutionary history of *Pseudogasteromyzon* species based on complete mitogenomes has not been fully established. This study presents an exploration of the features, structures, and the significant implications of mitochondrial genomes in *Pseudogasteromyzon* species. The total length of the 11 mitogenome sequences ranged from 16,561 bp to 16,574 bp. All but the *trnS1* gene exhibited the typical clover-leaf secondary structure among the 22 tRNAs. Cluster analysis utilizing the values of relative synonymous codon usage and phylogenetic analysis based on mitogenome sequences consistently yielded coherent topologies within the *Pseudogasteromyzon* species. Additionally, the Pleistocene epochs bore witness to a rapid differentiation event within the *Pseudogasteromyzon* genus. These findings present the first insights into the origin and phylogeny of *Pseudogasteromyzon* species.

**Abstract:**

As indicator organisms for water pollution detection, *Pseudogasteromyzon* species play a vital role in aquatic environment monitoring. We have successfully sequenced the mitogenomes of *P. fasciatus jiulongjiangensis* and *P. myersi* and downloaded the mitogenomes of nine other *Pseudogastromyzon* fish on GenBank to conduct a detailed comparative analysis of their phylogenetic relationships and evolutionary history. The findings revealed a conservation in both gene composition and gene order. Except for the *trnS1* gene lacking dihydrouracil arms, the other 21 tRNAs showed the typical clover-leaf secondary structure. According to the ΔRSCU method, we identified the seven most abundant optimal codons: CUA, GUA, CCA, CAA, GAA, AGC, and GGC. The construction of maximum parsimony, maximum likelihood, and Bayes trees yielded congruent topologies, and the 11 *Pseudogastromyzon* species were clustered into two major clusters. Among them, one of which was composed of *P. fangi*, *P. changtingensis changtingensis*, and *P. changtingensis tungpeiensis*, while the remaining eight species formed another cluster, further subdivided into five smaller clusters. Distinct clusters formed between *P. fasciatus jiulongjiangensis* and *P. meihuashanensis*, *P. cheni* and *P. peristictus*, and *P. laticeps* and *P. lianjiangensis*, and the remaining two species were clustered separately, thereby enhancing our understanding of them. Furthermore, our analysis results of divergence times revealed that these 11 *Pseudogasteromyzon* species underwent rapid differentiation in the Pleistocene epochs. Overall, our study sheds light on the phylogenetic relationship and evolutionary history of *Pseudogasteromyzon* species, providing a necessary knowledge foundation for further understanding the intricacies of an ecosystem health assessment.

## 1. Introduction

*Pseudogastromyzon* fish belongs to the family Gastromyzontidae and has 11 species, making them important biological indicator species [1,2,3]. These pint-sized benthic freshwater fish typically thrive in fast-flowing streams and are adept at attaching themselves to rocks using specialized pelvic fins, allowing them to cling to surfaces [4]. Additionally, the captivating appearance and distinct body coloration of *Pseudogastromyzon* fish have sparked interest in its potential as an aquarium fish in China. However, the nature habitat of this species is limited [5], and there is a notable lack of molecular research on it. This not only impedes a comprehensive understanding of *Pseudogastromyzon* species’ genetic information but also limits insights into its evolutionary history. Genetic information serves as the fundamental cornerstone and pivotal factor in species evolution. It shapes life characteristics, steers evolutionary paths, and preserves genetic diversity within populations [6]. To address these limitations and to ensure the rational development, utilization, and conservation of *Pseudogastromyzon* species’ natural resources, it is necessary to have a deep understanding of the genetic evolution of *Pseudogastromyzon* species.

In general, the typical structure of an animal mitogenome consists of a closed-circular molecule with a size ranging from 15 to 20 kb [7], comprising 22 tRNA genes, 13 protein-coding genes (PCGs), two rRNA genes, and a control region (CR) [8]. The mitochondrial genome has the advantages of strict matrilineal inheritance and self-replication, and it is not prone to genetic recombination in the process of generation transmission, and its evolution rate is faster than that of nuclear genome [9]. With the rapid advancement of molecular biotechnology, mitogenome molecular marker technology has found widespread applications in various fields including systems evolution, population genetics, and adaptive evolution [10,11,12]. However, the existing reports mainly focus on submitting mitochondrial genome sequences, and there is almost no information on the phylogenetic relationships and evolutionary history of these *Pseudogastromyzon* species [4,13,14].

Codon usage bias (CUB) is a prevalent phenomenon in nature, representing a form of molecular evolution [15]. CUB may vary greatly among different organisms, even within the genes of the same organism [16]. Further research on the usage patterns of codons in *Pseudogastromyzon* species can increase our comprehensive understanding of the molecular mechanism of their adaptation to the environment and help to discover the evolutionary relationship among them.

In this study, we sequenced the mitochondrial genomes of *P. fasciatus jiulongjiangensis* and *P. myersi*. We identified the predominant optimal codons in *Pseudogastromyzon* species, assessed their phylogenetic relationship, and estimated their evolutionary time. It is expected to provide help for a better understanding of the evolutionary process, population genetics, and phylogenetics of *Pseudoogastromyzon* species and other related species.

## 2. Materials and Methods

### 2.1. Samples Collection

*P. fasciatus jiulongjiangensis* and *P. myersi* were caught by fishermen in Kowloon Reservoir (22°21′5″ N, 114°9′32″ E) and Shing Mun Reservoir (22°22′54″ N, 114°9′4″ E) in the Hong Kong Special Administrative Region, China, respectively. They were purchased as experimental individuals and anesthetized with 60 mL/L MS-222, and then placed in 10 mL sampling tubes (Guangzhou Jet Bio-Filtration Co., Ltd., Guangzhou, China) containing anhydrous ethanol, and then deposited in the National Engineering Research Laboratory of Marine Biotechnology and Engineering, Ningbo University, with the catalog number of WC-153721. All the individuals were identified by Zheng and Li (1986) [5] and Tang and Chen (1996) [17] based on the previous taxonomic works. 

### 2.2. DNA Extraction, PCR Amplification, and Sequencing

Total DNA was extracted from the muscle tissue of the samples using Universal Genomic DNA Kit (CoWin Biosciences Co., Ltd., Jiangsu, China). The quality of DNA was assessed using 0.8% agarose gels, and the high-quality genomic DNA of *P. fasciatus jiulongjiangensis* and *P. myersi* was amplified as PCR template. The primers were designed based on *P. meihuashanensis* mitogenome (NC_046445.1), and the primer list for PCR amplification is shown in Table S1. The program began by a pre-cycling denaturation cycle at 94 °C for 4 min; 35 cycles of denaturation at 94 °C for 1 min, annealing at 58 °C for 30 s, extension at 72 °C for 1 min, and a post-cycling extension at 72 °C for 10 min. The quality of PCR products was evaluated by electrophoresis on 1.0% agarose gels, and the high-quality PCR products were sequenced by Sanger technology.

### 2.3. Complete Mitogenome Analysis

The mitogenome fragments of *P. fasciatus jiulongjiangensis* and *P. myersi* were spliced using NOVOPlasty 4.21 software [18], followed by manual inspection using the Seqman program in the Lasergene software [19]. Then, the assembled mitogenome sequences were subjected to functional annotation using the MITOS web server (https://mitos2.bioinf.uni-leipzig.de/index.py). The graphical representations of the complete mitogenomes were generated using OrganellarGenomeDRAW version 1.3.1 [20]. The secondary structures of tRNAs were identified using tRNAscan-SE version 2.0 [21]. Finally, the complete mitogenomes of *P. fasciatus jiulongjiangensis* and *P. myersi* were deposited in the GenBank database, and the accession numbers are BankIt2727481 (OR350601) and BankIt2728028 (OR353705), respectively.

### 2.4. Determination of Optimal Codons and Cluster Analysis

The GC content, including the total GC content (GC_all_), as well as the third base of the codon (GC_3_), the second base of the codon (GC_2_), and the first base of the codon (GC_1_), along with the effective number of codons (ENC) and codon adaption index (CAI), were calculated using the online program available at http://www.bioinformatics.nl/emboss-explorer/ (accessed on 7 August 2023). CAI serves as an indicator of the bias in synonymous codon usage within a gene [22,23], while ENC assesses the non-uniform utilization of synonymous codon groups [24]. Relative synonymous codon usage (RSCU) was calculated using CodonW version 1.4.2. The codon exhibiting a higher frequency in high-expression genes compared to low-expression genes is designated as the optimal codon, in accordance with the principles outlined by Ikemura (1985) [25]. Based on ENC values, the top 10% of genes (equivalent to two genes in this particular study) with the highest and lowest ENC values were categorized into the high-expression and low-expression groups, respectively. According to the ΔRSCU method, codons meeting the criteria of ΔRSCU > 0.08 and demonstrating an RSCU value exceeding 1 in the high-expression group while being below 1 in the low-expression group were identified as optimal codons. The RSCU value of each codon was calculated and obtained, except for AUG, UGG, and three stop codons (TAA, TAG, and TGA). Subsequently, the RSCU values of the remaining 59 codons were employed for cluster analysis using SPSS version 22.0. The heatmap of the RSCU was drawn using the online website CIMminer (http://discover.nci.nih.gov/cimminer/home.do (accessed on 7 August 2023).

### 2.5. Phylogenetic Analysis

Currently, there are 11 known species in the *Pseudogastromyzon* genus. In this study, we embarked on a comprehensive analysis of the phylogenetic relationships among the 11 *Pseudogastromyzon* species. Firstly, we utilized a collection of 33 well-established mitochondrial genome sequences from fishes within the Gastromyzontidae family, all readily accessible in GenBank. *Sinogastromyzon puliensis* (GenBank accession: AP011298.1) served as the designated outgroup. Our approach began with the compilation of the mitochondrial genome sequences, which were meticulously aligned using BioEdit version 7.0.9 with default settings [26]. Subsequently, these aligned sequences were seamlessly integrated using Sequence Matrix version 1.7.8 [27]. To identify the best-fit model for nucleotide substitution, we employed the Akaike information criterion score through jModeltest version 2 [28,29]. The selected model, GTR+I+G, was determined to be the most suitable. The construction of phylogenetic trees, central to our exploration, was accomplished using Mrbayes version 3.2.7a, employing Bayesian inference (BI) techniques introduced by Ronquist et al. (2012) [30]. The parameters settings were as follows: generations: 5,000,000; sampling frequency: 100; number of Markov chains: four; number of simultaneous runs: two; and burn-in fraction: 0.25. In tandem, we constructed the maximum likelihood (ML) tree using RAxML [31], with the GTR+GAMMA model identified as the optimal evolutionary framework, and the number of the bootstrap replicates was 1000. Additionally, we generated a maximum parsimony (MP) tree using PAUP version 4.0a167 (Swofford, 2002), offering an alternative perspective on the data. Confidence values for the system tree branch were substantiated by 1000 bootstrap values, and key metrics including the consistency index (CI), retention index (RI), tree length (TL), and rescaled consistency index (RC) were calculated to ensure rigorous analysis. Finally, the phylogenetic trees were visualized using Figtree version 1.4.3.

### 2.6. Divergence Times Estimation

The divergence times of *Pseudogastromyzon* species were estimated based on the mitogenome sequences. This was achieved by employing a relaxed uncorrelated lognormal clock, paired with a Yule speciation model as the tree prior, within the framework of BEAST version 1.8.4. For time correction, we referenced the differentiation times between *Liniparhomaloptera qiongzhongensis* and *Liniparhomaloptera disparis* (15.6 Mya), between *Vanmanenia pingchowensis* and *Vanmanenia hainanensis* (3.98 Mya) [32]. The operating parameters were set as follows: the Markov chain was executed in triplicate, each run spanning a total of 10,000,000 steps, with posterior samples drawn every 1000 steps, the model was set to GTR+I+G, and the initial 25% of samples were considered as burn-in and thus discarded. The software Tracer v.1.7 was used to view and detect the effective sample size of each parameter, ensuring that it was greater than 200. TreeAnnotator v.2.6.2 was used to estimate the upper and lower bounds of the 95% confidence interval for differentiation time. The analysis results were visualized using Figtree version 1.4.3.

## 3. Results and Discussion

### 3.1. Mitochondrial Genome Organization and Base Composition

We sequenced two complete mitogenomes of *P. fasciatus jiulongjiangensis* and *P. myersi*, and then downloaded the known mitogenomes of nine other *Pseudogastromyzon* species from the GenBank database, namely, *P. fangi* (MN123556.1), *P. cheni* (MZ853163.1), *P. laticeps* (MZ853164.1), *P. lianjiangensis* (MZ853166.1), *P. changtingensis changtingensis* (NC_046437.1), *P. changtingensis tungpeiensis* (NC_046438.1), *P. fasciatus fasciatus* (NC_046441.1), *P. meihuashanensis* (NC_046445.1), and *P. peristictus* (NC_046446.1). The total length of the 11 mitogenome sequences ranged from 16,561 bp (*P. fasciatus fasciatus*/*P. fasciatus jiulongjiangensis*/*P. meihuashanensis*) to 16,574 bp (*P. changtingensis tungpeiensis*). The complete mitogenomes of *P. fangi*, *P. changtingensis tungpeiensis*, and *P. changtingensis changtingensis* contained 13 protein-coding genes (PCGs), 22 transfer RNA genes (tRNAs), two ribosomal RNA genes (rRNAs), and two non-coding regions. Compared with these three species, the remaining eight *Pseudogastromyzon* species have an extra non-coding region in their mitochondrial genomes (Table S2). Further examination unveiled that within the 22 tRNAs (*trnQ*, *trnA*, *trnN*, *trnC*, *trnY*, *trnS2*, *trnE*, and *trnP*) and one of 13 PCGs (*nad6*) were encoded on the light strand (L-strand), while the remaining genes were encoded on the heavy strand (H-strand). The specific locations of these mitochondrial genes within the 11 *Pseudogastromyzon* species were meticulously illustrated in Figure S1 and Table S2. As previously reported, the distribution patterns of these genes were highly similar to those of other teleost fish [10,33]. In scrutinizing the complete mitochondrial genome organization across these 11 *Pseudogastromyzon* species, it emerged that the intergenic spacer regions of *P. fasciatus jiulongjiangensis* were 19, while those of the remaining 10 *Pseudogastromyzon* species were all 18, and the overlap regions of *P. fangi*, *P. changtingensis tungpeiensis* and *P. changtingensis changtingensis* were all 10, while those of the remaining 8 *Pseudogastromyzon* species were all 11. The mitogenome organizations among the 11 *Pseudogastromyzon* species were all closely aligned, with only a small amount of overlap regions among adjacent genes; it indicated that RNA transcription and protein translation of the 11 mitochondrial genomes may be more effective than these of their genomes. Moreover, the analysis results of base composition bias showed that the content of A+T in the 11 *Pseudogastromyzon* mitogenomes was higher than that of C+G, showing a strong A+T bias (54.10−55.03%). The base-skew of the 11 mitochondrial genome sequences was statistically analyzed, and the results showed that the AT-skew ([A−T]/[A+T]) value ranged from 0.0692 (*P. changtingensis changtingensis*) to 0.0860 (*P. laticeps*), and the GC-skew ([G−C]/[G+C]) value varied from −0.0274 (*P. peristictus*) to −0.248 (*P. changtingensis tungpeiensis*). These results indicated that the base composition of the 11 *Pseudogastromyzon* mitogenomes was strongly A-skewed and C-skewed. This similarity may be caused by the balance between mutation pressure and selection pressure during the replication process; base composition bias is a common phenomenon in teleost fishes, which reflects the conservatism of the 11 *Pseudogasteromyzon* mitogenomes in the evolution process [34,35].

### 3.2. Protein-Coding Genes

The sequence lengths of 13 tandem PCGs within the 11 *Pseudogastromyzon* mitogenomes ranged from 11,429 bp (*P. fasciatus jiulongjiangensis*) to 11,437 bp (*P. fangi*/*P. changtingensis tungpeiensis*/*P. changtingensis changtingensis*). The content of base composition of the 13 PCGs was different, among which the content of base A was the most, the content of base G was the least, and the content of A+T ranged from 53.41% (*P. myersi*) to 54.61% (*P. peristictus*). Moreover, the AT-skew and GC-skew values of the 13 PCGs in the 11 *Pseudogastromyzon* mitogenomes were shown in Table S3, a consistent pattern was observed in AT-skew values, with *nad1*, *nad2*, *cox2*, *atp8*, *nad4*, and *nad5* genes displaying positive values, and the GC-skew values for these genes were consistently negative values. An exception was noted with the *nad1* gene of *P. changtingensis tungpeiensis*, which exhibited a negative AT-skew value. Conversely, the *nad6* gene in all 11 *Pseudogastromyzon* mitogenomes showcased negative AT-skew values paired with positive GC-skew values. The remaining six genes featured negative AT-skew and GC-skew values, with the *cob* gene of *P. lianjiangensis* being the only exception, displaying a positive AT-skew value. In general, these characteristics were common in other teleost fishes [10], except for the above two cases. Additionally, the initiation codon for the *cox1* gene was uniquely GTG among the 13 PCGs, whereas the initiation codons for the remaining 12 PCGs were uniformly ATG. This characteristic was not exclusive to the 11 *Pseudogastromyzon* species, as it has been observed in other teleost fishes, such as *Cheilopogon doederleinii* [36], *Rivulus marmoratus* [37], *Beaufortia pingi* [38], and *Salvelinus gritzenkoi* [39]. In contrast to the stability exhibited by initiation codons, termination codons displayed greater variability within and between species. Specifically, four types of termination codons were identified among the 11 *Pseudogastromyzon* species: TAA, TAG, incomplete T-- and TA-. More detailed information on initiation and termination codons is presented in Table S2. Termination codons in fish mitogenomes have demonstrated rapid evolution and adaptability, as reported in prior studies [40,41]. The presence of incomplete termination codons was hypothesized to be a result of post-transcriptional polyadenylation, a common phenomenon observed in animal mitogenomes [8,42].

### 3.3. Transfer and Ribosomal RNA Genes

All tRNAs of the 11 *Pseudogastromyzon* mitogenomes were identified using tRNAscan-SE2.0 [21]. The total tRNAs lengths of *P. fangi*, *P. changtingensis tungpeiensis*, and *P. changtingensis changtingensis* were all 1559 bp, while the remaining eight *Pseudogastromyzon* species were all 1560 bp. Compared with the other eight *Pseudogastromyzon* species, the dihydrouridine loops of the *trnK* gene within *P. fangi*, *P. changtingensis tungpeiensis*, and *P. changtingensis changtingensis* mitogenomes displayed a reduction of one base “A” (Figure S2). In the 11 *Pseudogastromyzon* mitogenomes, the arrangement of tRNAs on both the heavy and light strands remained consistent, encompassing 14 tRNAs encoded on the H-strand and 8 tRNAs on the L-strand. This distribution pattern is a prevalent feature in many fish mitogenomes, akin to *Rasbora tornieri* [43], *Osteochilus salsburyi* [10], and *Rhynchocypris oxycephalus* [44]. Among the 22 tRNAs, the arrangement order of these genes was the same as that of vertebrate [7], and 21 tRNAs could be folded into the typical clover-leaf secondary structures, except for the *trnS1* gene lacking dihydrouracil arms. Compared with other mitochondrial genes, the nucleotide composition of these tRNAs across the 11 *Pseudogastromyzon* mitogenomes remained remarkably conserved and exhibited robust stability [7]. Notably, the anticodon loop of *trnT* and *trnV* genes was longer than that of the other tRNAs, with a length of 9 bp, and the gene characteristics were common in the mitochondrial genomes of most other Cyprinid fishes [10,12]. Furthermore, the relatively conserved tRNAs within these 11 *Pseudogastromyzon* mitogenomes revealed several noncanonical match or mismatch base pairs. G-U base pair mismatches were the most prevalent, ranging from 22 to 36 instances, followed by A-C base pair mismatches (7 to 12 instances). Mismatches involving A-A, C-C, and U-U base pairs ranged from 1 to 4 instances. Interestingly, while such mismatches were evident in *P. fasciatus jiulongjiangensis*, *P. fangi*, *P. fasciatus fasciatus* and *P. meihuashanensis*, they were absent in the other seven *Pseudogastromyzon* species (Table 1). These noncanonical nucleotide pairings were predominantly situated within the stems of DHU, TΨC, anticodon, and acceptor regions, with a predominant occurrence of G-U base pairs (Figure S2). These mismatched base pairs formed relatively weak bonds within the secondary tRNA structure across the 11 mitochondrial genomes. It is hypothesized that G-U base pair mismatches could be a widespread phenomenon in mitochondrial tRNAs, potentially corrected through post-transcriptional editing [45]. Furthermore, given the relatively limited genetic recombination of the mitochondrial genome during transmission, this phenomenon of base mismatch may play a role in mitigating the impact of deleterious gene mutations [46].

Within the realm of mitochondrial genomes, the *rrnS* and *rrnL* genes occupy a prominent position as extensively studied genes and are shared across organisms due to their common functional role. They contain both conserved and variable sequences, and their sequence changes are related to the evolutionary distance. It is now widely recognized that the two genes can be used as specific molecular markers to evaluate the genetic diversity and phylogenetic relationships of various organisms. There were many reports in this field, such as ectoparasites [47], alga [48,49], mitten crab [50], fish [51,52], etc. Here, we compared and analyzed the *rrnS* and *rrnL* genes within the 11 *Pseudogastromyzon* mitogenomes. The findings revealed that both *rrnS* and *rrnL* genes were located on the H-strand occupying the distinct position of *trnF-rrnS-trnV-rrnL-trnL2*. Both *rrnS* and *rrnL* genes had no spacer regions in their anterior and posterior positions, which were consistent with the typical characteristics of metazoa. The sequence length of the *rrnS* gene varied from 952 bp to 955 bp, while the sequence length of the *rrnL* sequence ranged from 1654 bp to 1660 bp. This observed variation in sequence length between the two rRNAs may potentially stem from inherent species differences [53]. Furthermore, a statistical examination of the base composition bias within the two rRNAs was undertaken. The C+G proportion of the *rrnS* gene fluctuated between 49.32% (*P. changtingensis changtingensis*) and 50.26% (*P. lianjiangensis*), while the C+G proportion of the *rrnL* gene fluctuated between 44.17% (*P. lattices*) and 45.44% (*P. fangi*). Both the *rrnS* and *rrnL* genes within the 11 *Pseudogastromyzon* mitogenomes exhibited negative GC-skew and positive AT-skew values, which indicated that the contents of adenines (As) and cytosines (Cs) in these two genes were high. The *rrnS* and *rrnL* genes in eukaryotes exhibited high conservatism, but there were some differences in the rRNA base composition of these 11 *Pseudogastromyzon* species, indicating that these two rRNA genes may be effective molecular markers for studying phylogenetic relationships and species evolution of *Pseudogastromyzon* species.

### 3.4. Noncoding Regions

While the control region, abundant in A+T bases, does not encode proteins, its pivotal role in regulating mitochondrial DNA replication and transcription is widely acknowledged. In the 11 *Pseudogastromyzon* mitogenomes, a discernible control region positioned between the *trnP* and *trnF* genes has been identified. The sequence length of the control region ranged from 882 bp (*P. changtingensis changtingensis*) to 903 bp (*P. changtingensis tungpeiensis*), with these sequences located on the H-strands. Compared with other fishes, there was no significant difference in the sequence length and A+T content of the control region among different species, except for the insertion/deletion of individual loci [54,55]. Similar to many vertebrates, the control region in the 11 *Pseudogastromyzon* species could be partitioned into three distinct domains. Firstly, the termination-associated sequence (TAS), also referred to as the hypervariable domain, was 221 bp in length and contained a sole relevant sequence responsible for controlling mitochondrial replication termination. This segment sequence was formed by the core sequence TACAT and its reverse complementary sequence ATGTA, which contained a 50-base thermostable stem-loop. Secondly, the central conserved domain, measuring a total of 106 bp, contained three conserved sequences, namely CSB-F, CSB-E and CSB-D. Among them, the key sequence of CSB-F was AGAGACCACC, which was considered as a symbol to distinguish the termination-associated sequence from the central conserved domain [56]. Furthermore, there was a widely recognized GTGGG-box in CSB-E, which was described by Lee et al. (1995) [57]. The key sequence AGGGGACAAATATCGTGGGGGGT of GTGGG-box in eight *Pseudogastromyzon* species was identified, except for AGGGACAATATATTGTGGGT in *P fasciatus fasciatus* and *P cheni* and AGGGACAATATTGAGGGT in *P. persistus*. For the key sequence of CSB-D, these 11 species were identical, and all were TATTACTGGCATCTG. Lastly, the conserved sequence block (CSB) contained three conserved sequences (CSB1, CSB2 and CSB3), among which the key sequences of CSB1, CSB2, and CSB3 were TTCATCATTAAAAGACATA, CAAACCCCCTTACCCC, and TGTCAAACCCCCGAAACCA, respectively, except for CAAACCCCCCTACCCC of CSB-2 in *P. changtingensis changtingensis *(Table 2). By comparing with other fishes, it was found that the 11 *Pseudogastromyzon* species were the same as most teleost fishes, and these key sequences were highly conserved and easily recognized.

The noncoding region within vertebrate mitochondrial genomes constitutes a compact fragment that plays a pivotal role in governing the replication and transcription of mitochondrial DNA. This region primarily resides between the replication initiation site of the L-strand and tRNA genes. Based on its structural composition and positional distribution, this region can be further categorized into intergenic spacer regions and overlapping regions. Ten overlapping regions were identified in the mitochondrial genomes of *P. fangi*, *P. changtingensis tungpeiensis* and *P. changtingensis changtingensis*, with a total sequence length of 32 bp and a maximum number of overlapping bases of 10 bp, which was located between *atp6* and *atp8* genes. The remaining eight *Pseudogastromyzon* species identified 11 overlapping regions in this region, and the number of overlapping bases in this extra overlapping region was 45 bp, which was located between the *trnE* and *OH*_1 genes. The intergenic spacer regions of *P. fasciatus jiulongjiangensis* were 19, while those of the remaining ten species were all 18, but the sequence length of the maximum intergenic spacer region between species was 31 bp, which was located between the *trnN* and *trnC* genes (Table S2). In this region, it could identify the sequence (*OL*) that initiated L-strand replication, and the sequence consisted of 10 bases to form a conservative stem-loop. In the mitochondrial genome of vertebrates, this stem-loop mainly regulated the replication of the L-strand [58].

### 3.5. Mitochondrial Gene Rearrangement

**Figure 1:**
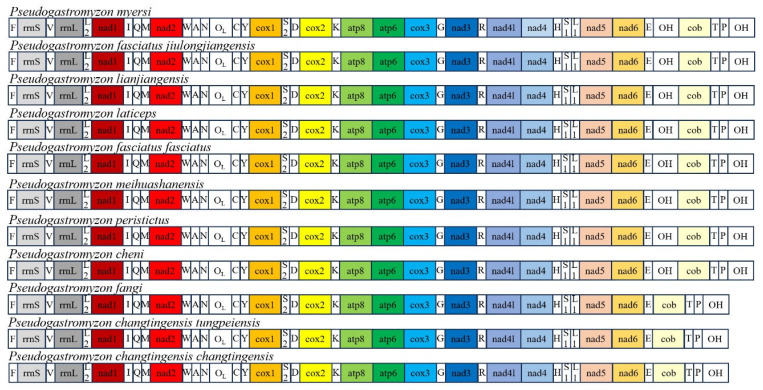
Gene arrangement of mitochondrial genomes from the genus *Pseudogasteromyzon*.

The gene arrangement order of *P. fangi*, *P. changtingensis tungpeiensis* and *P. changtingensis changtingensis* was the same as that of most teleost fishes. These results showed that the gene arrangement of the three *Pseudogastromyzon* species was conservative [59]. Of course, there were some groups of mitochondrial genes rearranged. Specifically, *P. myersi*, *P. fasciatus jiulongjiangensis*, *P. lianjiangensis*, *P. laticeps*, *P. fasciatus fasciatus*, *P. meihuashanensis*, *P. peristictus*, and *P. cheni* had an additional OH region located between the *trnE* and *cob* genes (Figure 1). These features also existed in some other organisms, for example, the mitochondrial gene order of *Muraenesox cinereus* was obviously rearranged, and the *nad6* and *trnE* genes were translocated to the location between the *trnT* and trnP genes, and one of the duplicated *D-loop* gene was translocated to the upstream of the *nad6* gene [60]. The mitochondrial genome of *Johnius grypotus* contained three noncoding sequences (NC1, NC2, and NC3) located between the *trnT* and *trnL_2_* genes [61]. These instances of gene rearrangement serve as potential models for exploring the mechanisms underlying mitochondrial instability in vertebrates.

### 3.6. Screening of Optimal Codons and Cluster Analysis

**Figure 2:**
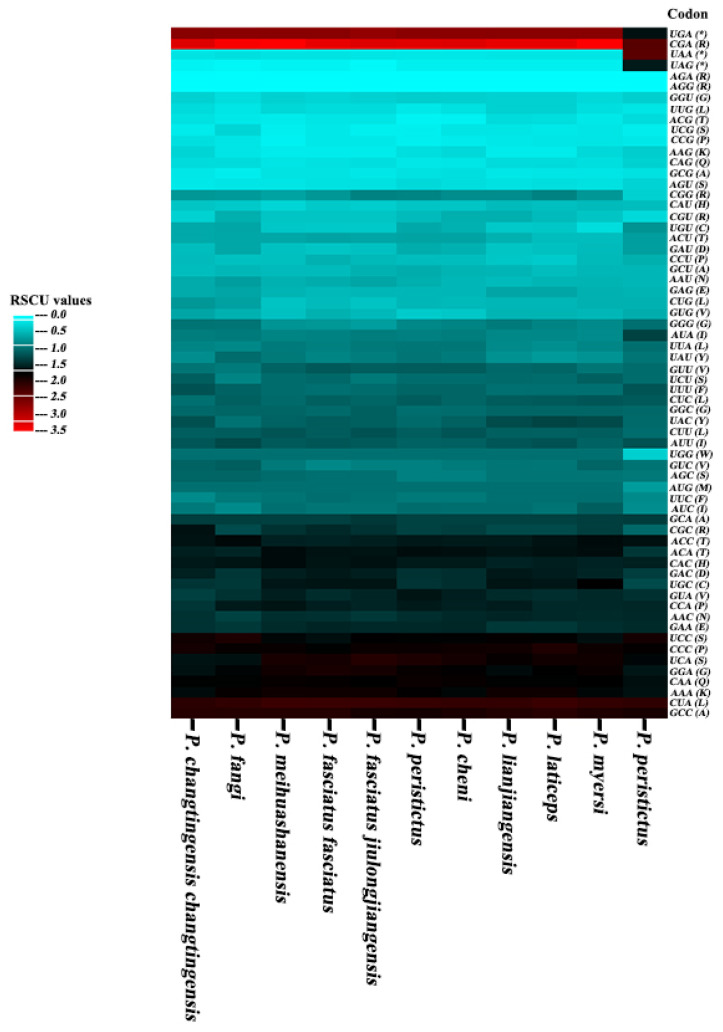
Heat map analysis of relative synonymous codon usage in the 13 PCGs of the 11 *Pseudogasteromyzon* species. The number scale on the left is the RSCU value.

**Figure 3:**
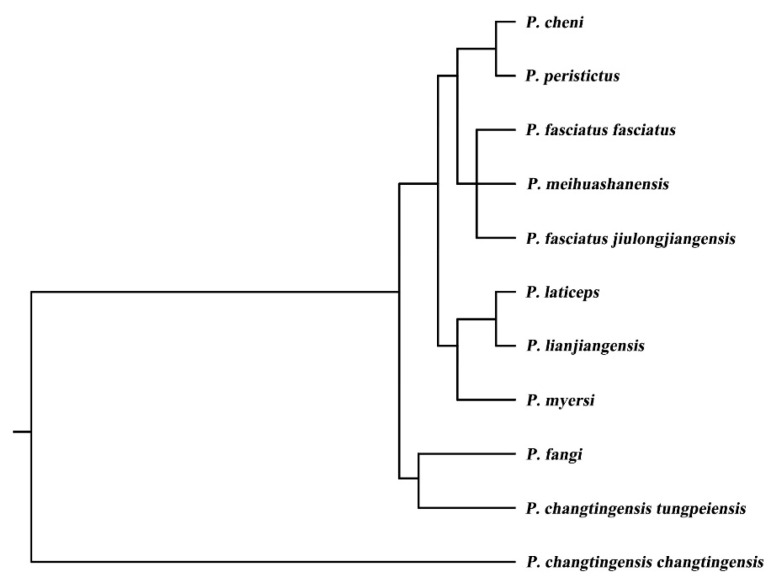
Schematic illustration of the cluster analysis based on the RSCU values of codons.

In this study, codon usage analysis was conducted on the 13 PCGs from the 11 *Pseudogasteromyzon* species. The values of CAI, ENC, GC_all_, GC_1_, GC_2_, and GC_3_ for each individual gene varied from 0.552 to 0.709, 28.473 to 56.399, 41.07% to 52.53%, 41.07% to 62.63%, 35.22% to 48.71%, and 32.14% to 51.52%, respectively. Generally, the order of GC content for different codon positions follows GC_1_ > GC_2_ > GC_3_. However, only 49 instances followed this order, while the GC content order for most genes was GC_1_ > GC_3_ > GC_2_. Of course, there were other variations in GC content orders. For example, the GC_1_ content of the *atp8* gene in *P. meihuashanensis*, *P. fasciatus fasciatus*, *P. changtingensis tungpeiensis*, and *P. cheni* was equal to their corresponding GC_2_ content, respectively. Similarly, the GC_2_ content of the *nad4l* gene in *P. fasciatus jiulongjiangensis*, *P. changtingensis changtingensis*, *P. lianjiangensis*, and *P. myersi* was equal to their corresponding GC_3_ content, respectively. The GC_2_ content of the *nad4* gene in *P. peristictus* was equal to its corresponding GC_3_ content (Table S4). Subsequently, the RSCU values were calculated to analyze codon usage patterns of different genes or gene groups. All PCG codons exhibited a significant bias, with RSCU values for NNA and NNC codons generally exceeding 1 (Figure 2), indicating their relatively frequent usage. RSCU is a crucial index that directly reflects codon usage bias [62]. From the perspective of the relative synonymous codon usage model, it can be observed that codons involving adenine and cytosine tend to be positioned in the third codon position among the synonymous substitution codons for each amino acid. Moreover, the high-expression and low-expression genes within the 11 *Pseudogasteromyzon* species were identified based on ENC values. Specifically, the high-expression and low-expression genes of *P. fasciatus jiulongjiangensis*, *P. cheni*, and *P. laticeps* were all *atp8* and *nad6* genes. Similar patterns were observed for *P. fasciatus fasciatus*, *P. meihuashanensis*, *P. lianjiangensis*, and *P. peristictus* (*atp6* and *nad6* genes), as well as for *P. fangi*, *P. changtingensis changtingensis*, and *P. changtingensis tungpeiensis* (*cox2* and *nad6* genes). Additionally, the *atp8* and *nad4l* genes were identified as the high-expression and low-expression genes for *P. myersi*. Furthermore, the optimal codons of these 11 *Pseudogasteromyzon* species were identified using the ΔRSCU method. The numbers of optimal codons in *P. fasciatus jiulongjiangensis*, *P. fangi*, *P. fasciatus fasciatus*, *P. meihuashanensis*, *P. changtingensis changtingensis*, *P. changtingensis tungpeiensis*, *P. cheni*, *P. laticeps*, *P. lianjiangensis*, *P. myersi*, and *P. peristictus* were 12, 15, 19, 18, 13, 14, 13, 11, 17, 10, and 21, respectively. In general, the most abundant optimal codons were CUA, GUA, CCA, CAA, GAA, AGC, and GGC, and all optimal codons are marked by asterisks in Table S5. A large number of studies showed that codon usage bias was related to many factors, which may be determined by the mutation or the combination of natural selection and mutation [63]. As a rule, the pressure of DNA sequence directed mutations and natural selection affecting gene translation were two key factors to explain differences in codon usage between species and within the genome. The results of this study indicated that mutations may not affect codon usage bias, except for natural selection. Additionally, a cluster analysis based on RSCU values was performed, revealing certain phylogenetic relationships among the *Pseudogasteromyzon* species. *P. fangi* was found to be closely related to *P. changtingensis changtingensis* and *P. changtingensis tungpeiensis*. *P. laticeps*, *P. lianjiangensis*, and *P. myersi* were grouped into one clade, and the phylogenetic relationship between *P. laticeps* and *P. lianjiangensis* was closer than that of *P. myersi*. *P. fasciatus fasciatus*, *P. meihuashanensis*, and *P. fasciatus jiulongjiangensis* were grouped into one clade; and *P. cheni* and *P. peristictus* were grouped into one clade (Figure 3).

### 3.7. Phylogenetic Analysis and Divergence Time Estimation

**Figure 4:**
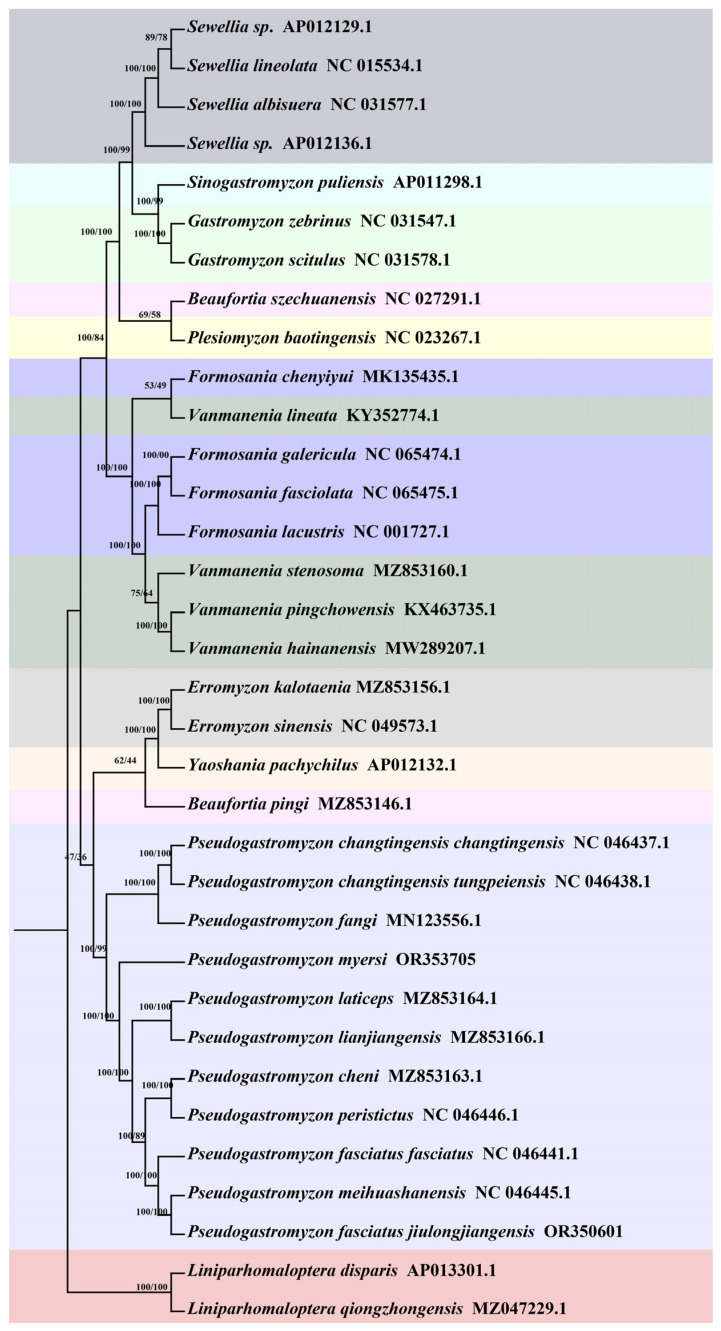
Phylogenetic tree of *Pseudogasteromyzon* based on the Bayesian and maximum likelihood analysis of 33 known mitogenome sequences of the family Gastromyzontidae. *Sinogastromyzon puliensis* was used as an outgroup. Numbers at each branch indicate Bayesian posterior probabilities (BPP)/maximum likelihood (ML) bootstrap values (%).

**Figure 5:**
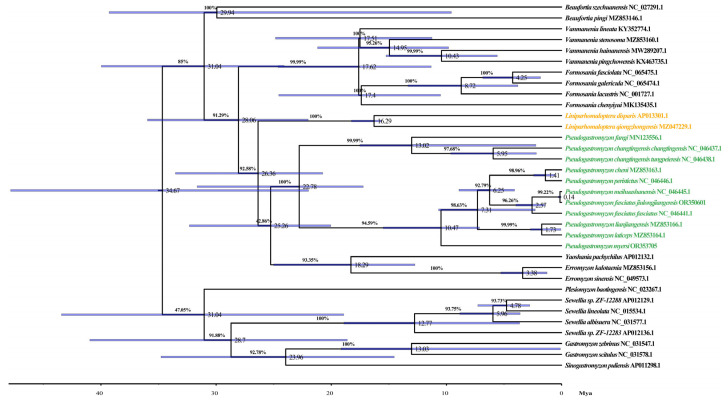
Divergence time estimates for *Pseudogasteromyzon* based on the complete mitochondrial genomes. The percentage on the node corresponds to the posterior probability of the node. Numbers in the nodes correspond to age estimates for the major clades. Blue bars indicate 95% highest posterior density intervals (HPD) for nodes of interest. Orange represents the calibration nodes of *Liniparhomaloptera qiongzhongensis* and *L. disparis*. Green represents the differentiation time node of the 11 *Pseudogastromyzon* species.

*Pseudogastromyzon* species, as regional small freshwater fish with high requirements for water quality, is one of the indicator organisms for assessing the degree of water pollution. Although they can play a certain warning role in environmental protection, their evolutionary history is still unknown, which is not conducive to the protection of their natural germplasm resources. In order to unravel the phylogenetic relationships and evolutionary history of *Pseudogastromyzon* species, we undertook comprehensive phylogenetic analyses and estimated divergence times using 33 known mitogenome sequences from the Gastromyzontidae family. In this study, the nucleotide sequences of the complete mitogenome were used to construct ML, BI, and MP trees. The phylogenetic tree included 15 genera of the family Gastromyzontidae, and the ML and BI analyses produced congruent branching patterns, with high ML bootstrap support and Bayesian posterior probability values (Figure 4). For MP analyses, TL was 28,153, CI was 0.360139 (0.322094), RI was 0.527390 (0.527390), and RC was 0.189934 (0.169869) for all sites and parsimony-informative sites (in parentheses) (Figure S3). These three phylogenetic trees exhibited concordant topological structures, highlighting a substantial clustering consistency among the 11 *Pseudogastromyzon* species. These results showed that the 11 *Pseudogastromyzon* species were clustered into two major clusters. Among them, one of which was composed of *P. fangi*, *P. changtingensis changtingensis*, and *P. changtingensis tungpeiensis*. Zheng and Li (1986) found that the mouthparts and number of vertical transverse spots of *P. changtingensis changtingensis* were very similar to those of *P. changtingensis tungpeiensis*. Presently, *P. changtingensis changtingensis* is only distributed in the upper reaches of the Han River. On the other hand, *P. changtensis tungpeiensis* is found in the Beijiang River, Jiulianshan Mountain Stream, and Rongjiang River in the eastern Pearl River system. Compared with the former two species, the mouthparts of *P. fangi* is similar to those of *P. changtingensis changtingensis*, and *P. changtingensis tungpeiensis*, but their body patterns and geographical distribution are different. *P. fangi* has some variation in body patterns in different distribution river sections. For example, specimens from Xijiang River have vertical spots, while those from Beijiang River (including Xiangjiang River) are mainly dotted with spots [5]. Combining their morphological characteristics and geographical distribution information, it can be seen that the phylogenetic relationship between *P. changtingensis changtingensis* and *P. changtingensis tungpeiensis* was relatively closer than that of *P. fangi*. The remaining eight species formed another cluster, further subdivided into five smaller clusters. Distinct clusters formed between *P. fasciatus jiulongjiangensis* and *P. meihuashanensis*, *P. cheni* and *P. peristictus*, and *P. laticeps* and *P. lianjiangensis*, signifying closer phylogenetic relationships within these pairs. The remaining two species were clustered separately. *P. fasciatus jiulongjiangensis* and *P. meihuashanensis* are distributed in the same water system, both of which belong to the Jiulong River [5,64]. *P. cheni* and *P. peristictus* are very similar in morphology, with smaller and denser round spots on the body surface. Because of the natural barrier formed by mountains and rivers, their distribution is limited, which may be one of the reasons for their relatively close phylogenetic relationship. Moreover, *P. fasciatus fasciatus* is mainly distributed in the Min River and the Ji River. *P. myersi* has irregular horizontal spots on its body surface, with several rows of dotted stripes on each fin, and on each side behind the linear ridges of the upper and lower lips, there is a harder fleshy or raised cushion like object, which is not found in other species of this genus *Pseudogastromyzon* [5]. This confirmed its distribution position in our phylogenetic relationship analysis. Overall, *P. fasciatus jiulongjiangensis*, *P. meihuashanensis*, and *P. fasciatus fasciatus* were first clustered into a cluster; the phylogenetic relationship between *P. fasciatus jiulongjiangensis* and *P. meihuashanensis* was closer than that of *P. fasciatus fasciatus*, and then these three *Pseudogastromyzon* species were clustered into a larger cluster with *P. cheni* and *P. peristictus*. Moreover, *P. laticeps* and *P. lianjiangensis* were clustered into a larger cluster with these five *Pseudogastromyzon* species, and finally these seven *Pseudogastromyzon* species and *P. myersi* form a major cluster. For another major cluster, the phylogenetic relationship between *P. changtingensis changtingensis*, and *P. changtingensis tungpeiensis* was closer than that of *P. fangi*. In these three phylogenetic trees, the topological structures of these 11 *Pseudogastromyzon* species clusters were completely consistent, with their posterior probabilities and bootstrap values at high levels. Furthermore, our phylogenetic analysis results were consistent with Chen et al.’s research results based on mitochondrial PCGs and rRNA genes, proving that our results were reliable [2]. Additionally, as shown in Figure 5, our analysis results of divergence times revealed that the genera *Liniparhomaloptera* and *Pseudogastromyzon* diverged about 26.36 million years ago (Mya) with 95% highest posterior density intervals (HPD) of 20.74–33.53 Mya, around the Oligocene (23.3–32.0 Mya). The divergence time of *P. fangi* was estimated to be approximately 13.02 Mya (95% HPD: 12.34–17.52), occurring mainly in middle Miocene, while that of *P. changtingensis changtingensis* and *P. changtingensis tungpeiensis* diverged approximately 5.95 Mya (95% HPD: 2.19–9.63), occurring mainly in late Miocene. The divergence time of *P. myersi* was estimated to be approximately 10.47 Mya (95% HPD: 7.14–15.48), around the late Miocene, while that of *P. laticeps* and *P. lianjiangensis* diverged approximately 1.73 Mya (95% HPD: 0.06–2.77), around the Pleistocene. The divergence time of *P. fasciatus fasciatus* was estimated to be approximately 6.25 Mya (95% HPD: 4.09–8.91), around the late Miocene, while that of the remaining four species was in Pleistocene. From the time axis, global cooling and polar ice increase occurred in the early Oligocene, and this process continued until the warming event in the late Oligocene. Subsequently, the amount of global ice remained at a low level, and the water temperature at the bottom of the ocean rose slightly. This trend reached its peak in the middle Miocene. The warming event in this period was global, and the temperature was even higher than now. Since then, the cooling event occurred again in the late Miocene, and the Antarctic ice sheet was formed again. The chronological context of these divergence times suggested that the differentiation of *Pseudogastromyzon* species coincided with global climatic events. The formation of Antarctic ice sheets during cooling events in the late Miocene and Pliocene influenced the evolutionary patterns of these species. The Pleistocene epochs witnessed significant fish adaptations to changing environments, which likely contributed to species formation and radiation evolution within *Pseudogastromyzon*. Overall, the divergence times of *Pseudogastromyzon* species exhibited parallels with those of many other teleost species, such as *Pampus argenteus* [65] and *Osteochilus salsburyi* [10]. To sum up, our study is the first to conduct a comprehensive analysis of the 11 *Pseudogastromyzon* mitogenomes. This valuable data revealed their phylogenetic relationships and evolutionary history, providing a necessary foundation for future research on the role of these organisms as indicators of water resource pollution.

## 4. Conclusions

In this study, we have successfully sequenced the mitogenomes of *P. fasciatus jiulongjiangensis* and *P. myersi* and conducted a detailed comparative analysis of phylogenetic relationships and evolutionary history of the 11 *Pseudogastromyzon* species. The total length of the 11 mitogenome sequences ranged from 16,561 bp to 16,574 bp. All but the *trnS1* gene exhibited the typical clover-leaf secondary structure among the 22 tRNAs. An exception was noted with the *nad1* gene of *P. changtingensis tungpeiensis*, which exhibited a negative AT-skew value. Compared with the other eight *Pseudogastromyzon* species, the dihydrouridine loops of the *trnK* gene within *P. fangi*, *P. changtingensis tungpeiensis*, and *P. changtingensis changtingensis* mitogenomes displayed a reduction of one base “A”. In noncanonical match or mismatch base pairs, A-A, C-C, and U-U base pairs were evident in the 22 tRNAs of *P. fasciatus jiulongjiangensis*, *P. fangi*, *P. fasciatus fasciatus*, and *P. meihuashanensis*; they were absent in the other seven *Pseudogastromyzon* species. Next, seven most abundant optimal codons of the 11 *Pseudogastromyzon* species were identified: CUA, GUA, CCA, CAA, GAA, AGC, and GGC. The construction of maximum parsimony, maximum likelihood, and Bayes trees yielded congruent topologies among the *Pseudogasteromyzon* species. The 11 *Pseudogastromyzon* species were clustered into two major clusters. Among them, one was composed of *P. fangi*, *P. changtingensis changtingensis*, and *P. changtingensis tungpeiensis*, while the remaining eight species formed another cluster, further subdivided into five smaller clusters. Distinct clusters formed between *P. fasciatus jiulongjiangensis* and *P. meihuashanensis*, *P. cheni* and *P. peristictus*, and *P. laticeps* and *P. lianjiangensis*, and the remaining two species were clustered separately. The Pleistocene epochs witnessed an early and rapid differentiation within the genus *Pseudogasteromyzon*, with major lineages diversifying over a relatively narrow timescale. Among them, the differentiation time of most *Pseudogasteromyzon* species was mainly concentrated within 10 Mya, mainly occurring in the Miocene and Pliocene. The differentiation time of *P. laticeps* and *P. lianjiangensis* was relatively recent, about 1.73 Mya, mainly occurring during the Pleistocene. Moreover, the newly sequenced *Pseudogasteromyzon* mitogenomes in this study will enhance our understanding of their mitochondrial genome structure and phylogenetic studies, providing basic information for further understanding why they can become a good candidate for studies related to environmental quality and condition sensitivity.

## Figures and Tables

**Table 1:** Noncanonical match base pairs of 22 tRNAs in the 11 *Pseudogastromyzon* mitogenomes.

Species	Noncanonical Match Base Pairs					
	A-A	A-C	C-C	C-U	G-U	U-U
*P. fasciatus jiulongjiangensis*	2	12	1	1	22	3
*P. fangi*	3	8	2	1	31	3
*P. fasciatus fasciatus*	2	11	1	1	24	3
*P. meihuashanensis*	2	12	1	1	24	3
*P. changtingensis changtingensis*	4	7	2	−	35	2
*P. changtingensis tungpeiensis*	3	8	2	−	36	3
*P. cheni*	2	11	2	−	26	3
*P. laticeps*	2	10	2	−	24	3
*P. lianjiangensis*	2	9	2	−	26	3
*P. myersi*	2	9	2	−	27	3
*P. peristictus*	2	12	2	−	26	3

**Table 2:** Sequence features of control region in the 11 *Pseudogastromyzon* mitogenomes.

Species	Termination-Associated Sequence (TAS)	Central Conserved Domain (CCD)			Conserved Sequence Block (CSB)		
		CSB-F	CSB-E	CSB-D	CSB-1	CSB-2	CSB-3
*P. fasciatus jiulongjiangensis*	TACATCTATGTAATATCACCAA	ATGTAGTAAGAGACCACC	AGGGACAATAATCGTGGGGGT	TATTACTGGCATCTG	TTCATCATTAAAAGACATA	CAAACCCCCTTACCCCC	TGTCAAACCCCGAAACCA
*P. fangi*	TACATATATGTATTATCACCATT	ATGTAGTAAGAGACCACC	AGGGACAATAATCGTGGGGGT	TATTACTGGCATCTG	TTCATCATTAAAAGACATA	CAAACCCCCTTACCCCC	TGTCAAACCCCGAAACCA
*P. fasciatus fasciatus*	TACATCTATGTAATATCACCAA	ATGTAGTAAGAGACCACC	AGGGACAATAATTGTGGGGGT	TATTACTGGCATCTG	TTCATCATTAAAAGACATA	CAAACCCCCTTACCCCC	TGTCAAACCCCGAAACCA
*P. meihuashanensis*	TACATCTATGTAATATCACCAA	ATGTAGTAAGAGACCACC	AGGGACAATAATCGTGGGGGT	TATTACTGGCATCTG	TTCATCATTAAAAGACATA	CAAACCCCCTTACCCCC	TGTCAAACCCCGAAACCA
*P. changtingensis changtingensis*	TACATATATGTATTATCACCAT	ATGTAGTAAGAGACCACC	AGGGACAATAATCGTGGGGGT	TATTACTGGCATCTG	TTCATCATTAAAAGACATA	CAAACCCCCCTACCCCC	TGTCAAACCCCGAAACCA
*P. changtingensis tungpeiensis*	TACATATATGTATTATCACCAT	ATGTAGTAAGAGACCACC	AGGGACAATAATCGTGGGGGT	TATTACTGGCATCTG	TTCATCATTAAAAGACATA	CAAACCCCCTTACCCCC	TGTCAAACCCCGAAACCA
*P. cheni*	TACATATATGTAATATCACCAA	ATGTAGTAAGAGACCACC	AGGGACAATAATTGTGGGGGT	TATTACTGGCATCTG	TTCATCATTAAAAGACATA	CAAACCCCCTTACCCCC	TGTCAAACCCCGAAACCA
*P. laticeps*	TACATTCATGTAATATCACCAA	ATGTAGTAAGAGACCACC	AGGGACAATAATCGTGGGGGT	TATTACTGGCATCTG	TTCATCATTAAAAGACATA	CAAACCCCCTTACCCCC	TGTCAAACCCCGAAACCA
*P. lianjiangensis*	TACATTCATGTAATATCACCAA	ATGTAGTAAGAGACCACC	AGGGACAATAATCGTGGGGGT	TATTACTGGCATCTG	TTCATCATTAAAAGACATA	CAAACCCCCTTACCCCC	TGTCAAACCCCGAAACCA
*P. myersi*	TACATACATGTATTATCACCAA	ATGTAGTAAGAGACCACC	AGGGACAATAATCGTGGGGGT	TATTACTGGCATCTG	TTCATCATTAAAAGACATA	CAAACCCCCTTACCCCC	TGTCAAACCCCGAAACCA
*P. peristictus*	TACATATATGTAATATCACCAA	ATGTAGTAAGAGACCACC	AGGGACAATAATTGTGAGGGT	TATTACTGGCATCTG	TTCATCATTAAAAGACATA	CAAACCCCCTTACCCCC	TGTCAAACCCCGAAACCA

Note: The control region was divided into three main regions, bounded by CSB-F and CSB-1, which were the termination-associated sequence (TAS), central conserved domain (CSB-F, CSB-E, CSB-D), and conserved sequence block (CSB-1, CSB-2, CSB-3). Among them, the key sequence of CSB-F was AGAGACCACC, which was considered as a symbol to distinguish the termination-associated sequence from central conserved domain, CSB-E could be identified through the GTGGG-box sequence, which was located upstream of CSB-D. The conserved sequence block (CSB) contained three conserved sequences (CSB1, CSB2 and CSB3).

## Data Availability

The sequence data have been submitted to GenBank under accession numbers BankIt2727481 (OR350601) and BankIt2728028 (OR353705).

## Supplementary Materials

The following supporting information can be downloaded at: https://www.mdpi.com/article/10.3390/ani14030495/s1. Table S1: List of primers for PCR amplification; Table S2: Summary of complete mitochondrial gene/element features of 11 Pseudogastromyzon species; Table S3: Description of base bias of 13 protein-coding genes in the 11 Pseudogastromyzon mitogenomes; Table S4: Codon adaption index (CAI), effective number of codon (ENC) and GC content of the 13 PCGs in the 11 Pseudogasteromyzon species; Table S5: Optimal codons analysis of the 13 mitochondrial protein coding genes in the 11 Pseudogasteromyzon species; Figure S1: Complete mitochondrial genomes of the 11 Pseudogasteromyzon species; Figure S2: Schematic diagram of tRNAs secondary structures and OL in the Pseudogasteromyzon mitogenomes; Figure S3: The maximum parsimony analysis of *Pseudogasteromyzon* genera based on the combined analysis of all known mitochondrial genome sequences of the family Gastromyzontidae from the NCBI database.
