# Supplementary material for: Comprehensive Analysis of Phylogenetic Relationship and Optimal Codons in Mitochondrial Genomes of the Genus Pseudogastromyzon
Source: Animals (Basel). 2024 Feb 2;14(3):495. doi: 10.3390/ani14030495 (PMC10854560; doi:10.3390/ani14030495)
Supplement: Supplementary file 1 [file animals-14-00495-s001.zip › Figure S1.pdf]

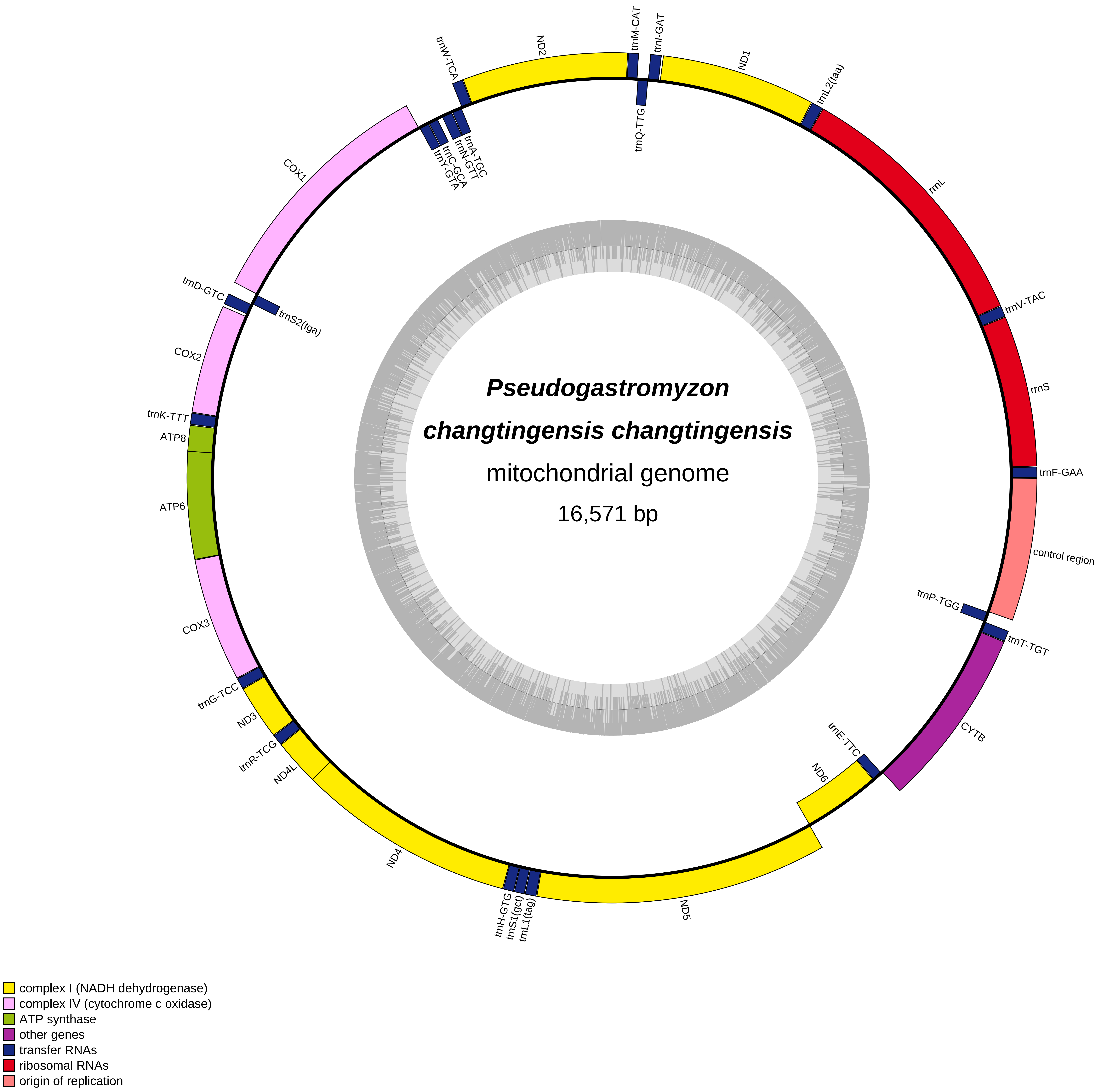
Pseudogastromyzon
changtingensis changtingensis
mitochondrial genome
16,571 bp
trnM-CAT
trnI-GAT
ND2
ND1
trnW-TCA
trnL2(taa)
trnQ-TTG
trnA-TGC
trnN-GTT
trnC-GCA
trnY-GTA
COX1
rrnL
trnD-GTC
trnS2(tga)
trnV-TAC
COX2
rrnS
trnK-TTT
ATP8
trnF-GAA
ATP6
control region
trnP-TGG
COX3
trnT-TGT
trnG-TCC
ND3
CYTB
trnE-TTC
trnR-TCG
ND4L
ND6
ND4
trnH-GTG
trnS1(gct)
trnL1(tag)
ND5
complex I (NADH dehydrogenase)
complex IV (cytochrome c oxidase)
ATP synthase
other genes
transfer RNAs
ribosomal RNAs
origin of replication

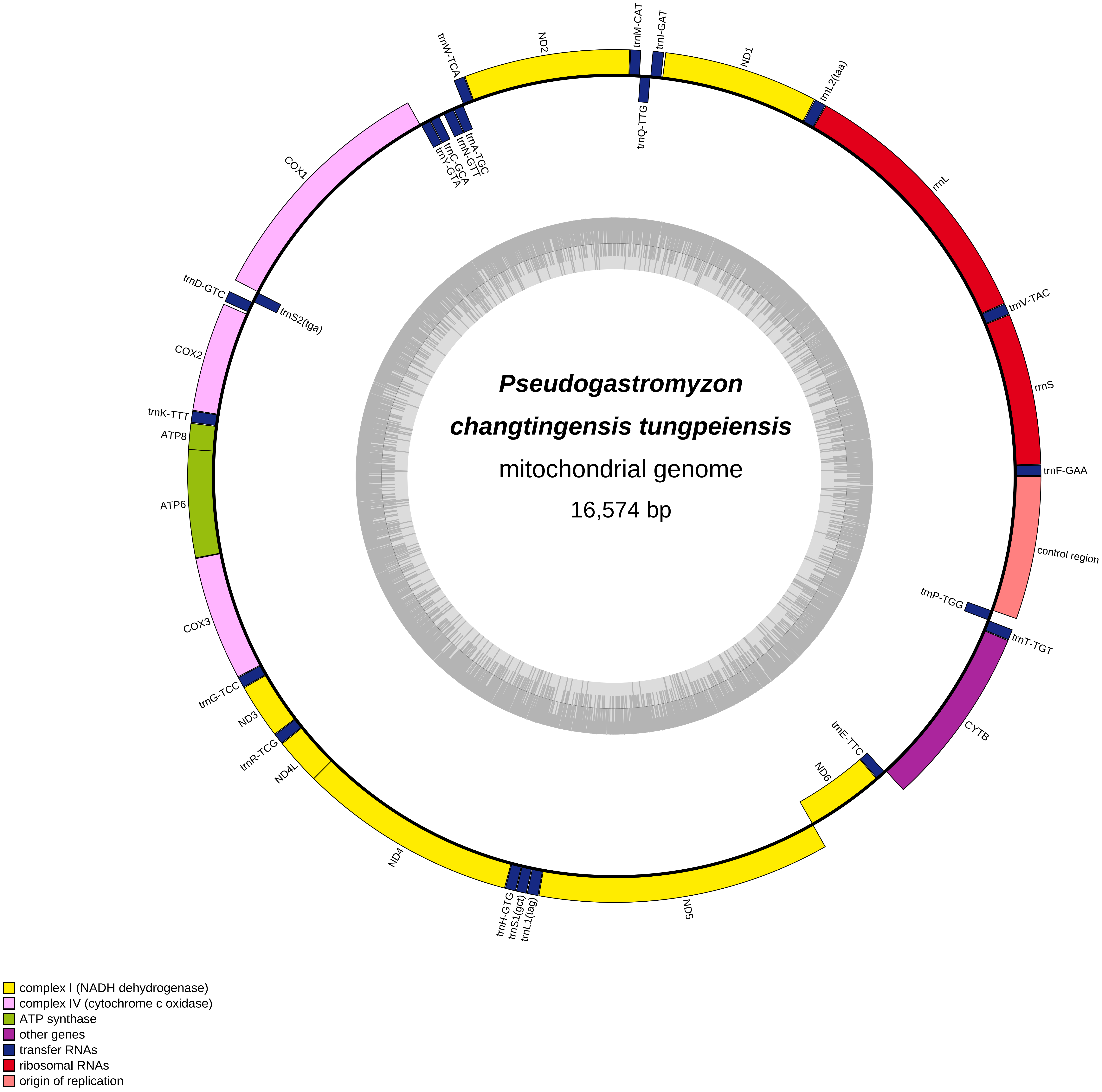
Pseudogastromyzon
changtingensis tungpeiensis
mitochondrial genome
16,574 bp
trnM-CAT
trnI-GAT
ND2
ND1
trnW-TCA
trnL2(taa)
trnQ-TTG
trnA-TGC
trnN-GTT
trnC-GCA
trnY-GTA
COX1
rrnL
trnD-GTC
trnS2(tga)
trnV-TAC
COX2
rrnS
trnK-TTT
ATP8
trnF-GAA
ATP6
control region
trnP-TGG
COX3
trnT-TGT
trnG-TCC
ND3
CYTB
trnE-TTC
trnR-TCG
ND4L
ND6
ND4
trnH-GTG
trnS1(gct)
trnL1(tag)
ND5
complex I (NADH dehydrogenase)
complex IV (cytochrome c oxidase)
ATP synthase
other genes
transfer RNAs
ribosomal RNAs
origin of replication

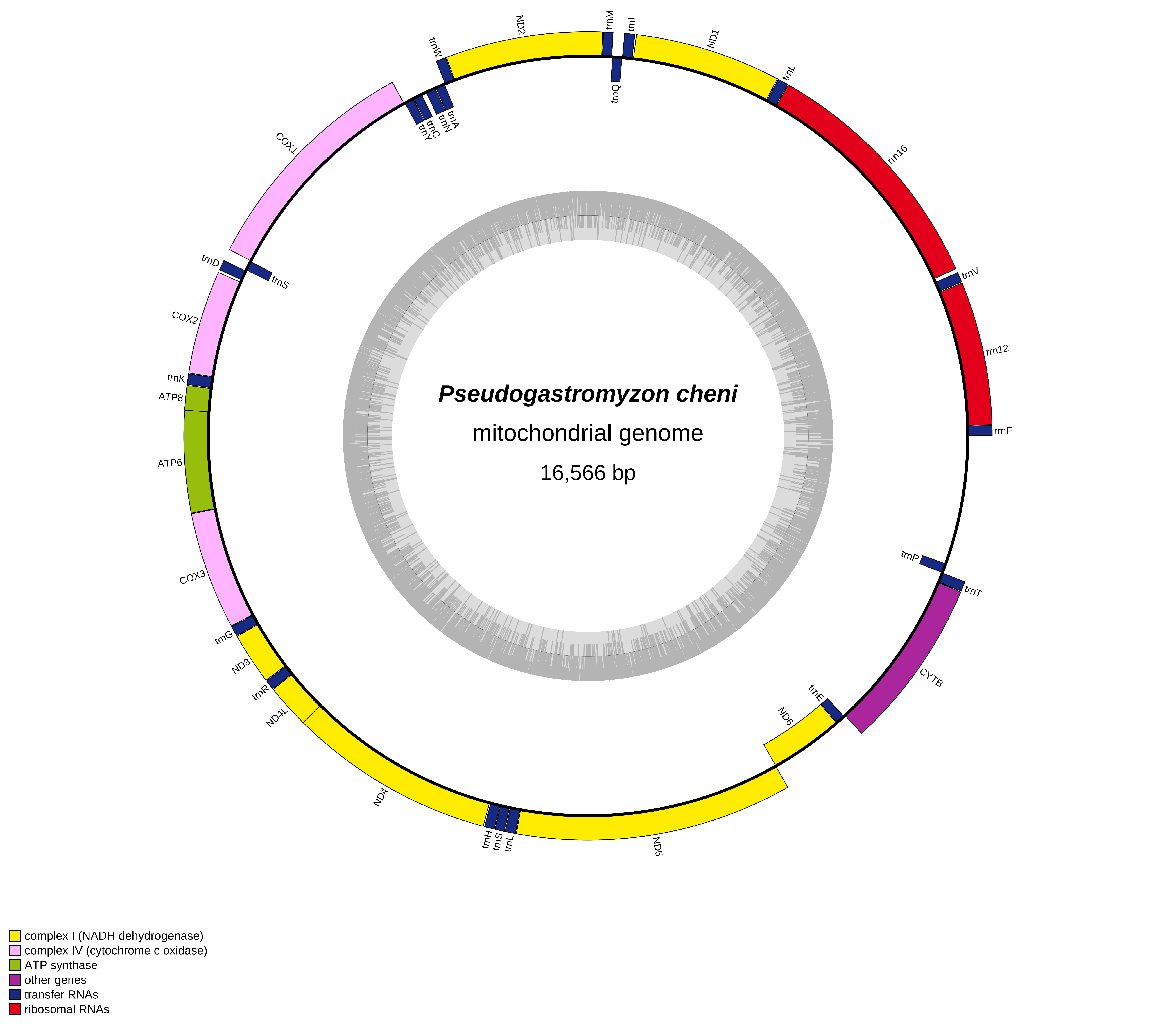

Pseudogastromyzon cheni
mitochondrial genome
16,566 bp
ND2
trnM
trnI
ND1
trnW
trnQ
trnL
trnA
trnN
trnC
trnY
COX1
rrn16
trnD
trnS
trnV
COX2
rrn12
trnK
ATP8
trnF
ATP6
trnP
trnT
COX3
trnG
CYTB
ND3
trnE
trnR
ND6
ND4L
ND4
trnH
trnS
trnL
ND5
complex I (NADH dehydrogenase)
complex IV (cytochrome c oxidase)
ATP synthase
other genes
transfer RNAs
ribosomal RNAs

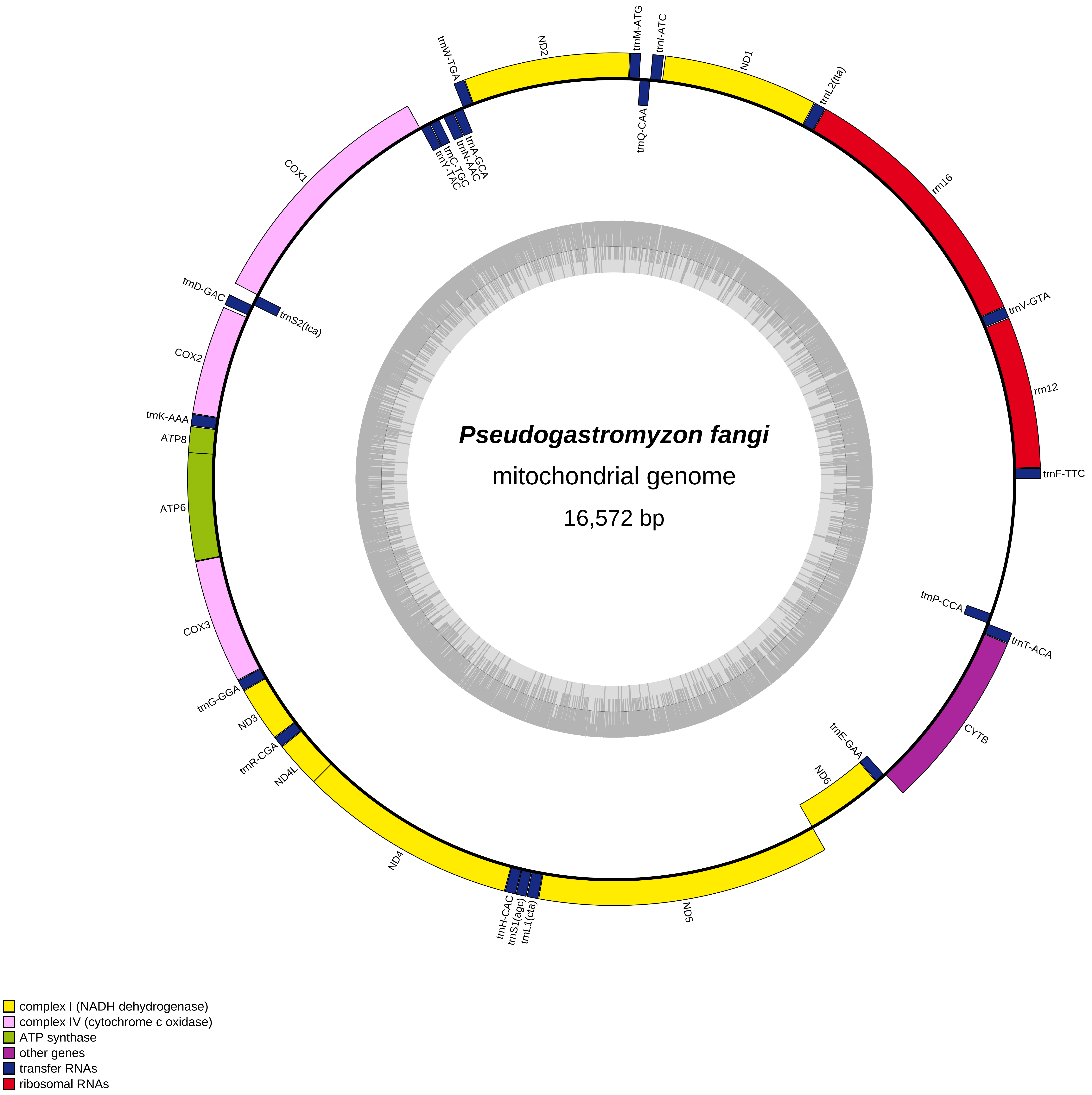

Pseudogastromyzon fangi
mitochondrial genome
16,572 bp
trnM-ATG
trnI-ATC
ND2
ND1
trnW-TGA
trnL2(tta)
trnQ-CAA
trnA-GCA
trnN-AAC
trnC-TGC
trnY-TAC
COX1
rrn16
trnD-GAC
trnS2(tca)
trnV-GTA
COX2
rrn12
trnK-AAA
ATP8
trnF-TTC
ATP6
COX3
trnP-CCA
trnT-ACA
trnG-GGA
ND3
CYTB
trnE-GAA
trnR-CGA
ND6
ND4L
ND4
trnH-CAC
trnS1(agc)
trnL1(cta)
ND5
complex I (NADH dehydrogenase)
complex IV (cytochrome c oxidase)
ATP synthase
other genes
transfer RNAs
ribosomal RNAs

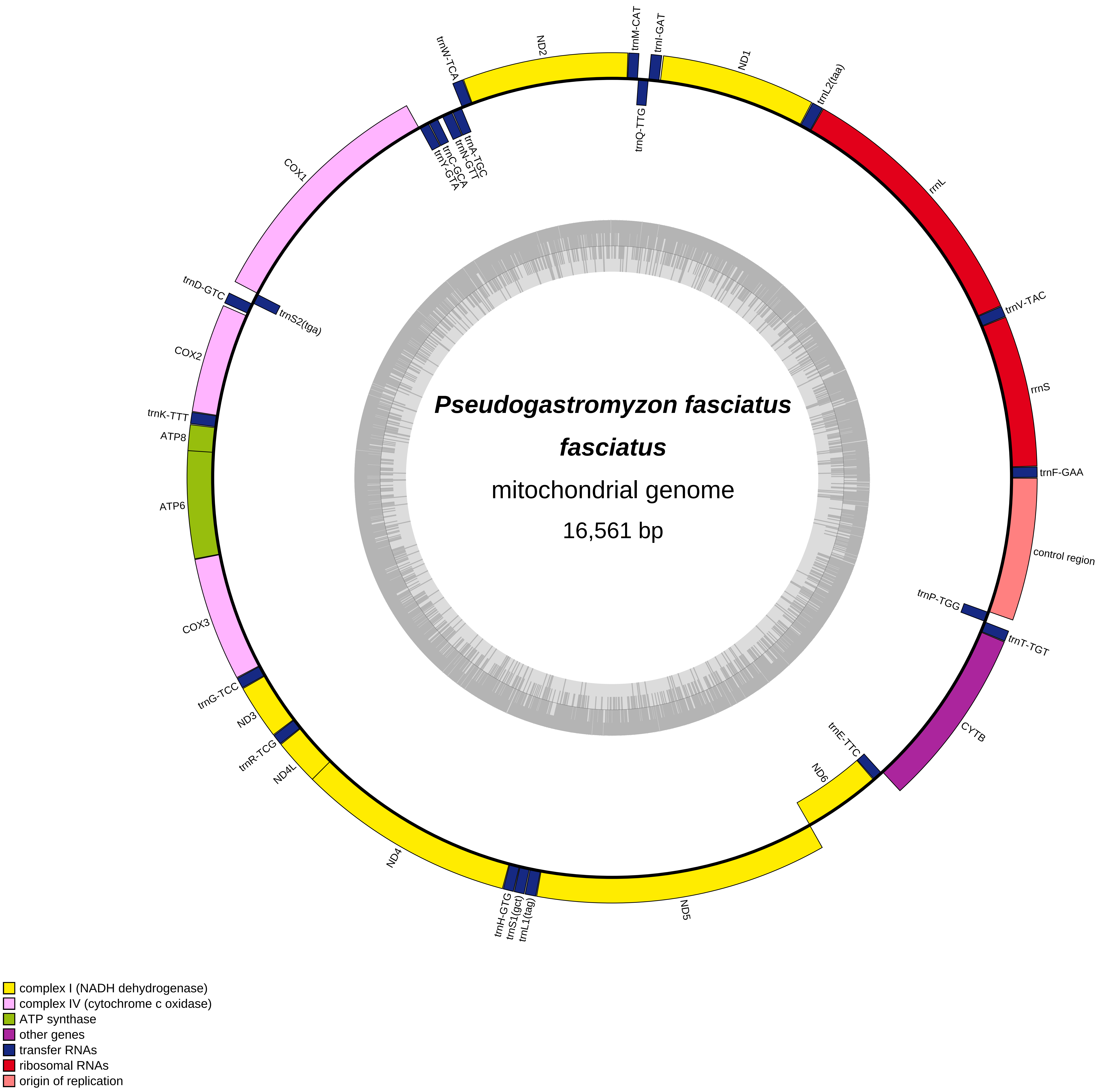

Pseudogastromyzon fasciatus fasciatus
mitochondrial genome
16,561 bp
trnM-CAT
trnI-GAT
ND2
trnW-TCA
ND1
trnL2(taa)
trnQ-TTG
trnA-TGC
trnN-GTT
trnC-GCA
trnY-GTA
COX1
rrnL
trnV-TAC
trnD-GTC
trnS2(tga)
COX2
rrnS
trnK-TTT
ATP8
trnF-GAA
ATP6
control region
trnP-TGG
trnT-TGT
COX3
trnG-TCC
ND3
CYTB
trnE-TTC
trnR-TCG
ND4L
ND6
ND4
trnH-GTG
trnS1(gct)
trnL1(tag)
ND5
complex I (NADH dehydrogenase)
complex IV (cytochrome c oxidase)
ATP synthase
other genes
transfer RNAs
ribosomal RNAs
origin of replication

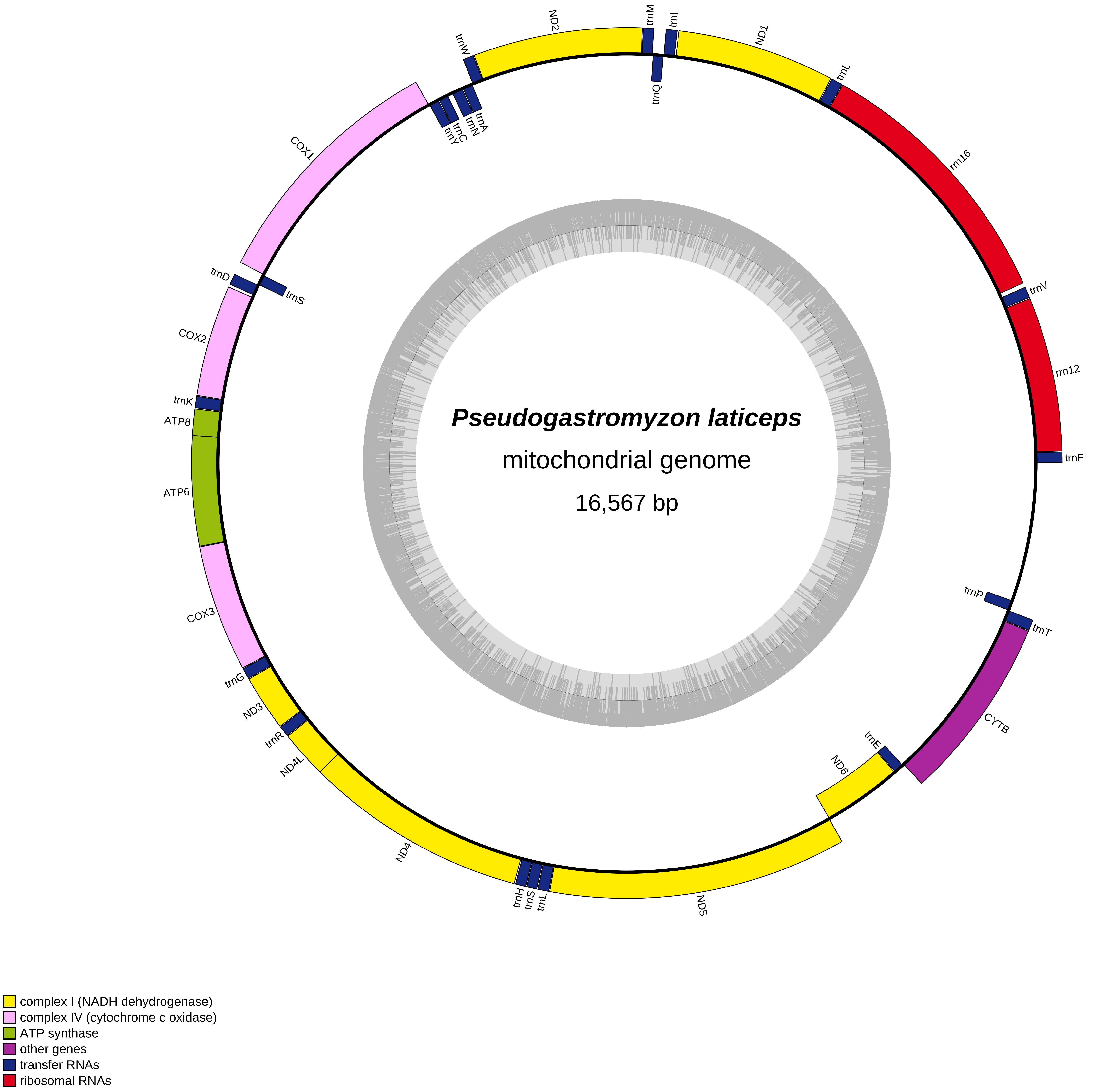
Pseudogastromyzon laticeps
mitochondrial genome
16,567 bp
trnM
trnI
ND2
ND1
trnW
trnQ
trnL
trnA
trnN
trnC
trnY
COX1
rrn16
trnD
trnS
trnV
COX2
rrn12
trnK
ATP8
trnF
ATP6
trnP
trnT
COX3
trnG
CYTB
ND3
trnE
trnR
ND6
ND4L
ND4
trnH
trnS
trnL
ND5
complex I (NADH dehydrogenase)
complex IV (cytochrome c oxidase)
ATP synthase
other genes
transfer RNAs
ribosomal RNAs

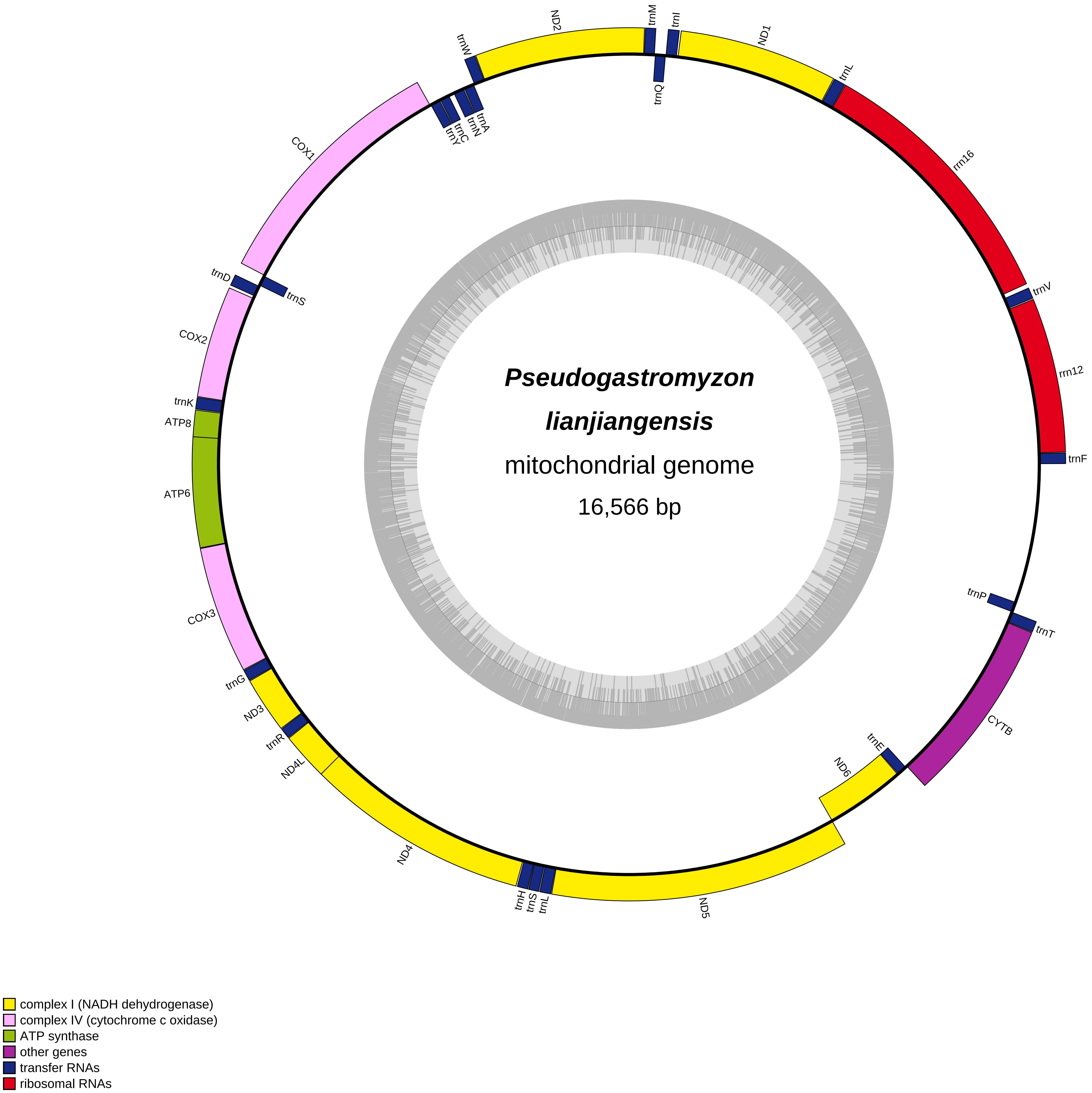

Pseudogastromyzon
lianjiangensis
mitochondrial genome
16,566 bp
trnM
trnI
ND2
ND1
trnW
trnQ
trnL
trnA
trnN
trnC
trnY
COX1
rrn16
trnD
trnS
trnV
COX2
rrn12
trnK
ATP8
trnF
ATP6
trnP
trnT
COX3
trnG
CYTB
ND3
trnE
trnR
ND6
ND4L
ND4
trnH
trnS
trnL
ND5
complex I (NADH dehydrogenase)
complex IV (cytochrome c oxidase)
ATP synthase
other genes
transfer RNAs
ribosomal RNAs

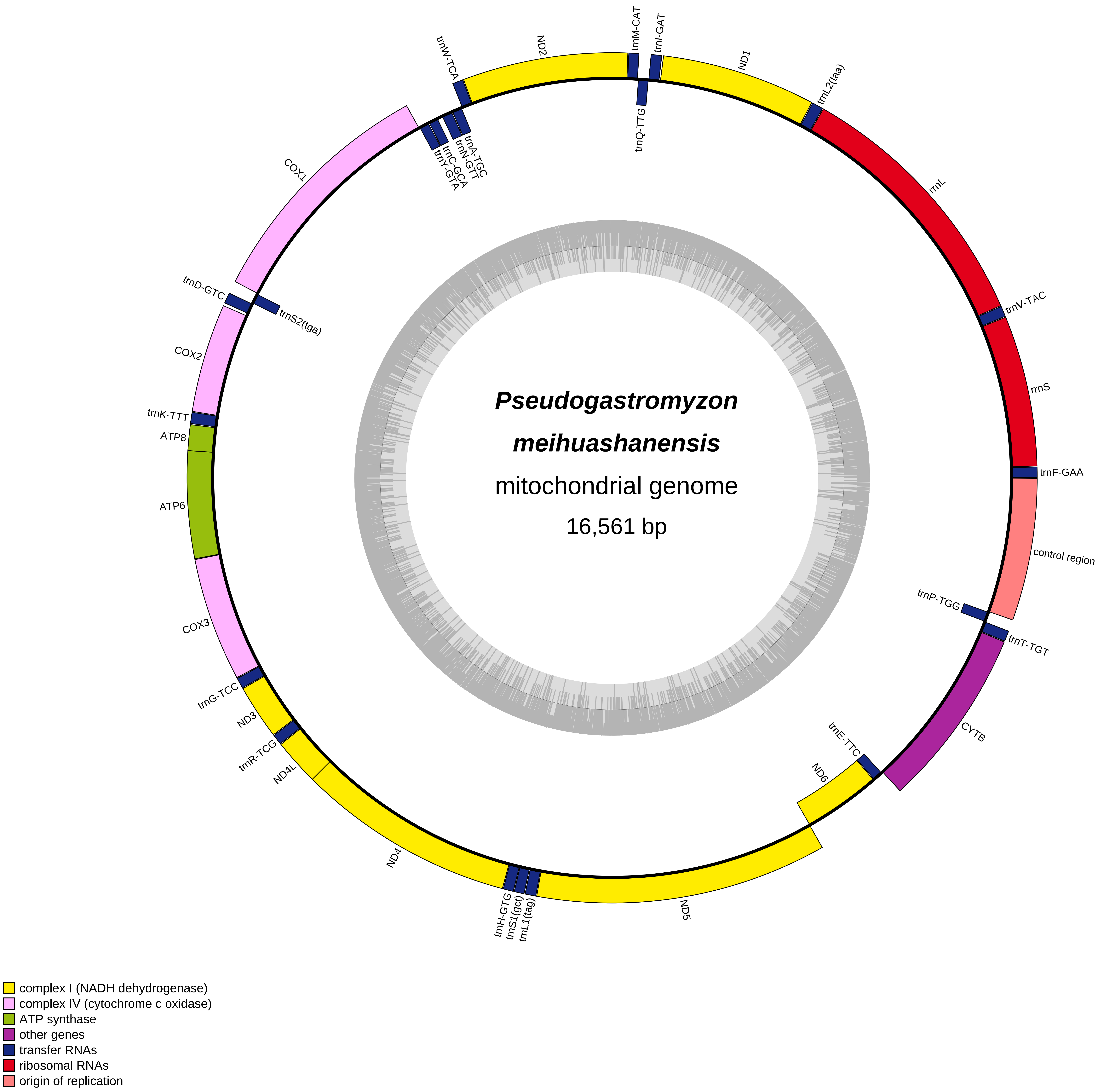

Pseudogastromyzon meihuashanensis
mitochondrial genome
16,561 bp
trnM-CAT
trnI-GAT
trnQ-TTG
ND2
trnW-TCA
trnA-TGC
trnN-GTT
trnC-GCA
trnY-GTA
ND1
trnL2(taa)
rrnL
trnV-TAC
rrnS
trnF-GAA
control region
trnP-TGG
trnT-TGT
CYTB
trnE-TTC
ND6
ND5
trnH-GTG
trnS1(gct)
trnL1(tag)
ND4
ND4L
trnR-TCG
ND3
trnG-TCC
COX3
ATP6
ATP8
trnK-TTT
COX2
trnD-GTC
trnS2(tga)
COX1
complex I (NADH dehydrogenase)
complex IV (cytochrome c oxidase)
ATP synthase
other genes
transfer RNAs
ribosomal RNAs
origin of replication

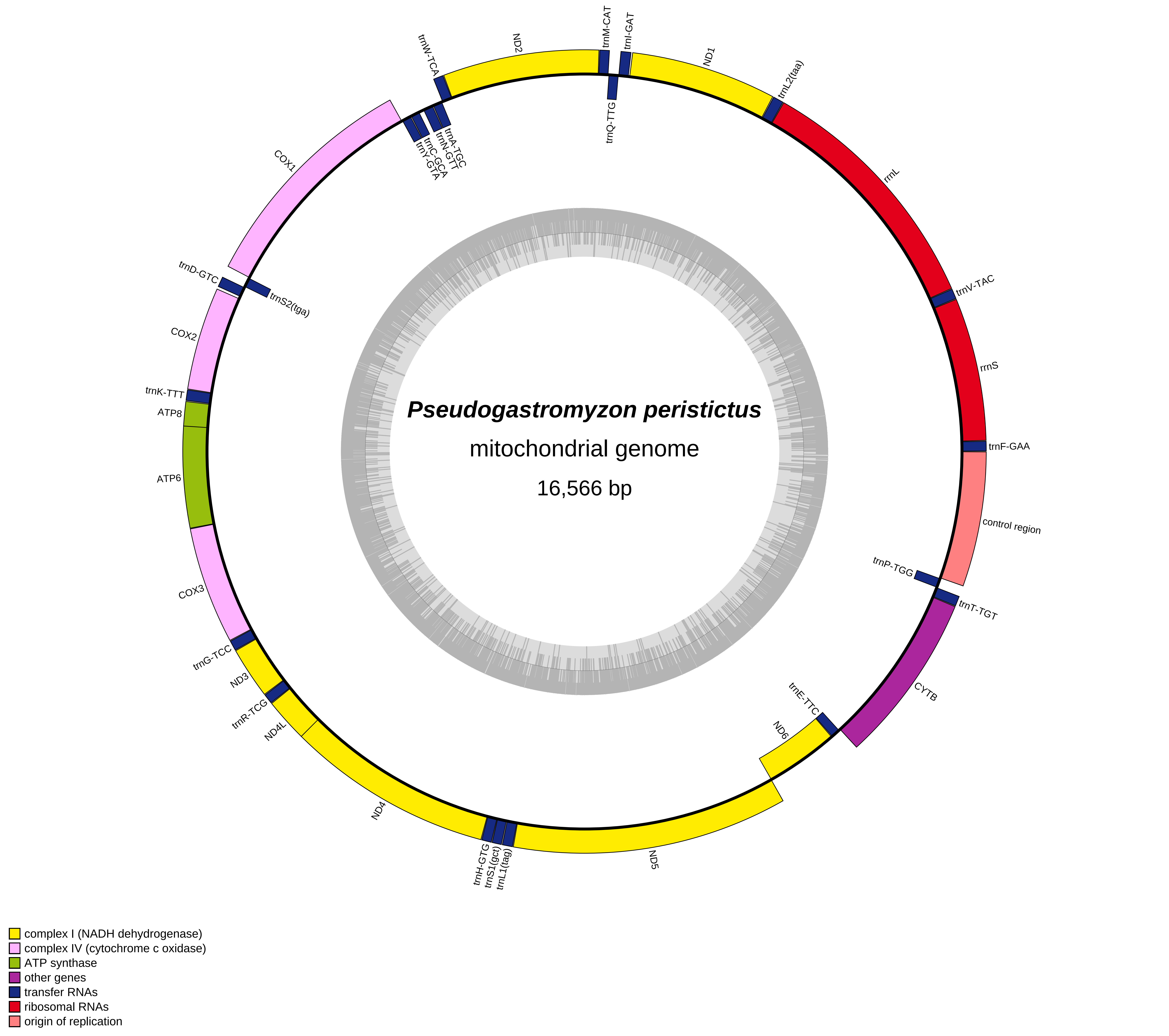

Pseudogastromyzon peristictus
mitochondrial genome
16,566 bp
trnM-CAT
trnI-GAT
ND2
ND1
trnW-TCA
trnL2(taa)
trnQ-TTG
trnA-TGC
trnN-GTT
trnC-GCA
trnY-GTA
COX1
rrnL
trnD-GTC
trnS2(tga)
trnV-TAC
COX2
rrnS
trnK-TTT
ATP8
trnF-GAA
ATP6
control region
trnP-TGG
trnT-TGT
COX3
trnG-TCC
CYTB
ND3
trnE-TTC
trnR-TCG
ND6
ND4L
ND4
trnH-GTG
trnS1(gct)
trnL1(tag)
ND5
complex I (NADH dehydrogenase)
complex IV (cytochrome c oxidase)
ATP synthase
other genes
transfer RNAs
ribosomal RNAs
origin of replication

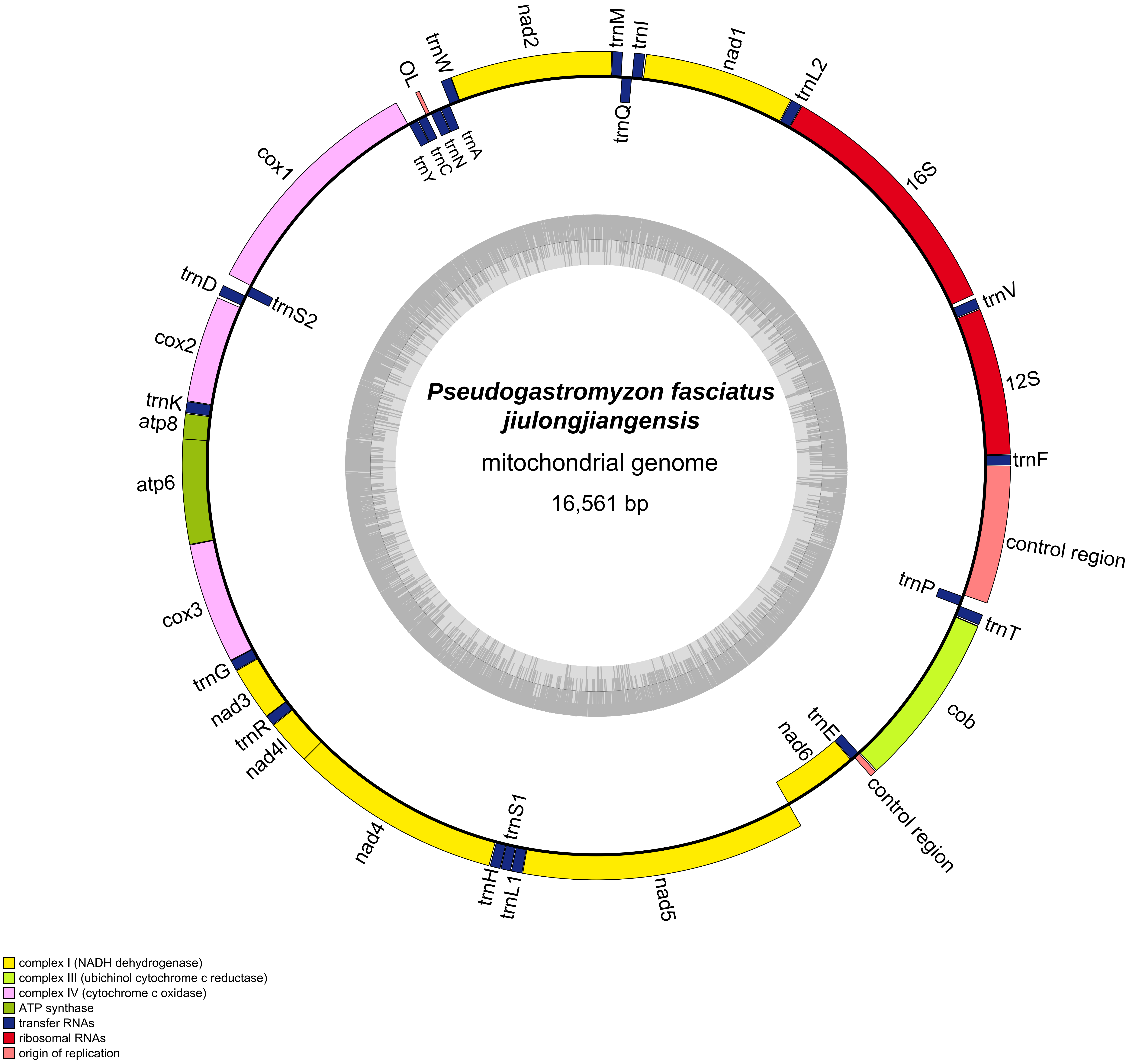
Pseudogastromyzon fasciatus jiulongjiangensis
mitochondrial genome
16,561 bp
nad2
trnM
trnI
nad1
trnL2
16S
trnV
12S
trnF
control region
trnP
trnT
cob
control region
trnE
nad6
nad5
trnS1
trnL1
trnH
nad4
nad4l
trnR
nad3
trnG
cox3
atp6
atp8
trnK
cox2
trnS2
trnD
cox1
OL
trnW
trnQ
trnA
trnN
trnC
trnY
complex I (NADH dehydrogenase)
complex III (ubichinol cytochrome c reductase)
complex IV (cytochrome c oxidase)
ATP synthase
transfer RNAs
ribosomal RNAs
origin of replication

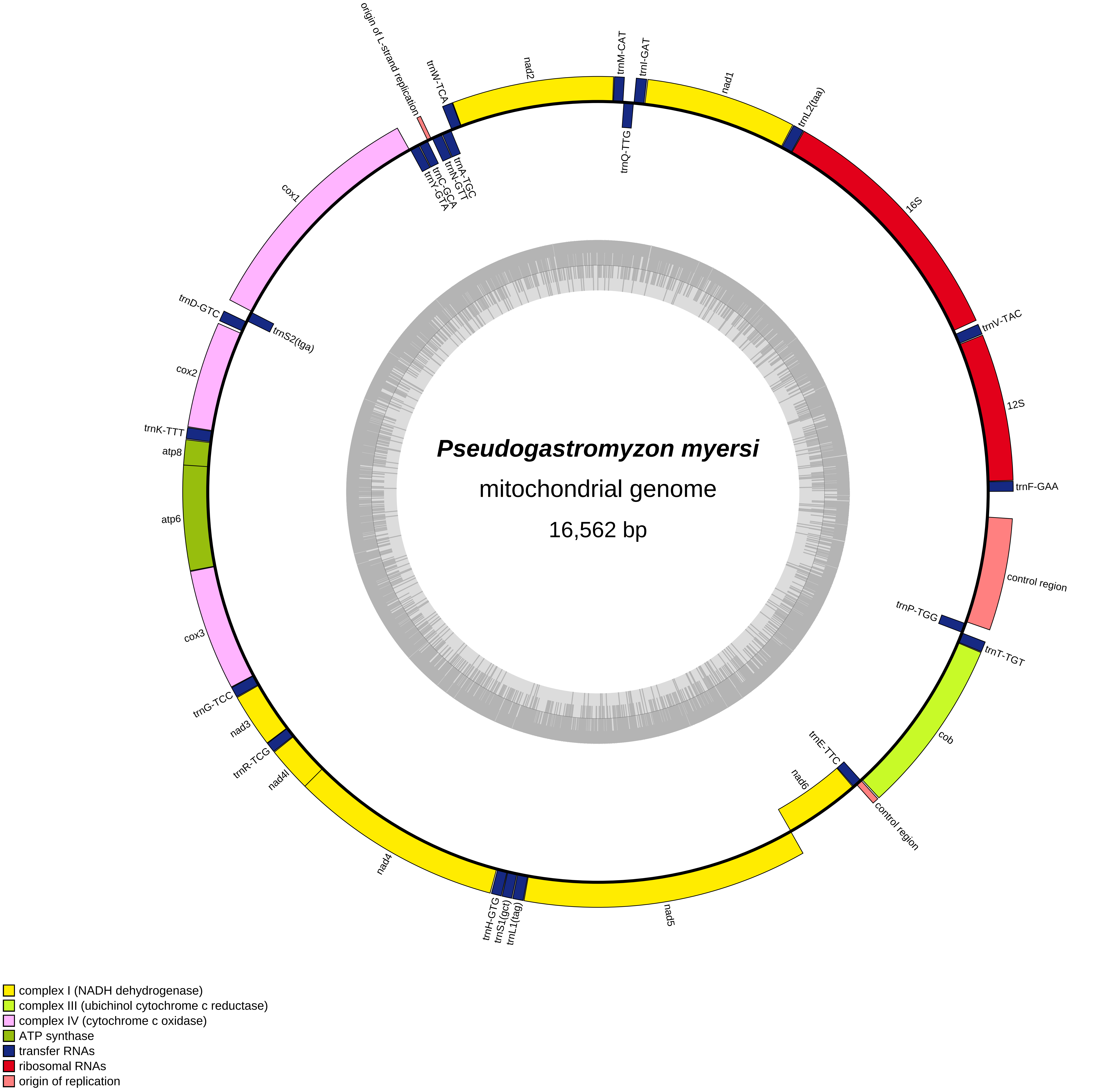

Pseudogastromyzon myersi
mitochondrial genome
16,562 bp
nad2
trnM-CAT
trnI-GAT
nad1
trnL2(taa)
16S
trnV-TAC
12S
trnF-GAA
control region
trnP-TGG
trnT-TGT
cob
control region
trnE-TTC
nad6
nad5
trnL1(tag)
trnS1(gct)
trnH-GTG
nad4
nad4l
trnR-TCG
nad3
trnG-TCC
cox3
atp6
atp8
trnK-TTT
cox2
trnD-GTC
trnS2(tga)
cox1
trnY-GTA
trnC-GCA
trnN-GTT
trnA-TGC
trnW-TCA
origin of L-strand replication
trnQ-TTG
complex I (NADH dehydrogenase)
complex III (ubichinol cytochrome c reductase)
complex IV (cytochrome c oxidase)
ATP synthase
transfer RNAs
ribosomal RNAs
origin of replication
