# Supplementary material for: Comprehensive Analysis of Phylogenetic Relationship and Optimal Codons in Mitochondrial Genomes of the Genus Pseudogastromyzon
Source: Animals (Basel). 2024 Feb 2;14(3):495. doi: 10.3390/ani14030495 (PMC10854560; doi:10.3390/ani14030495)

P. fasciatus jiulongjiangensis:

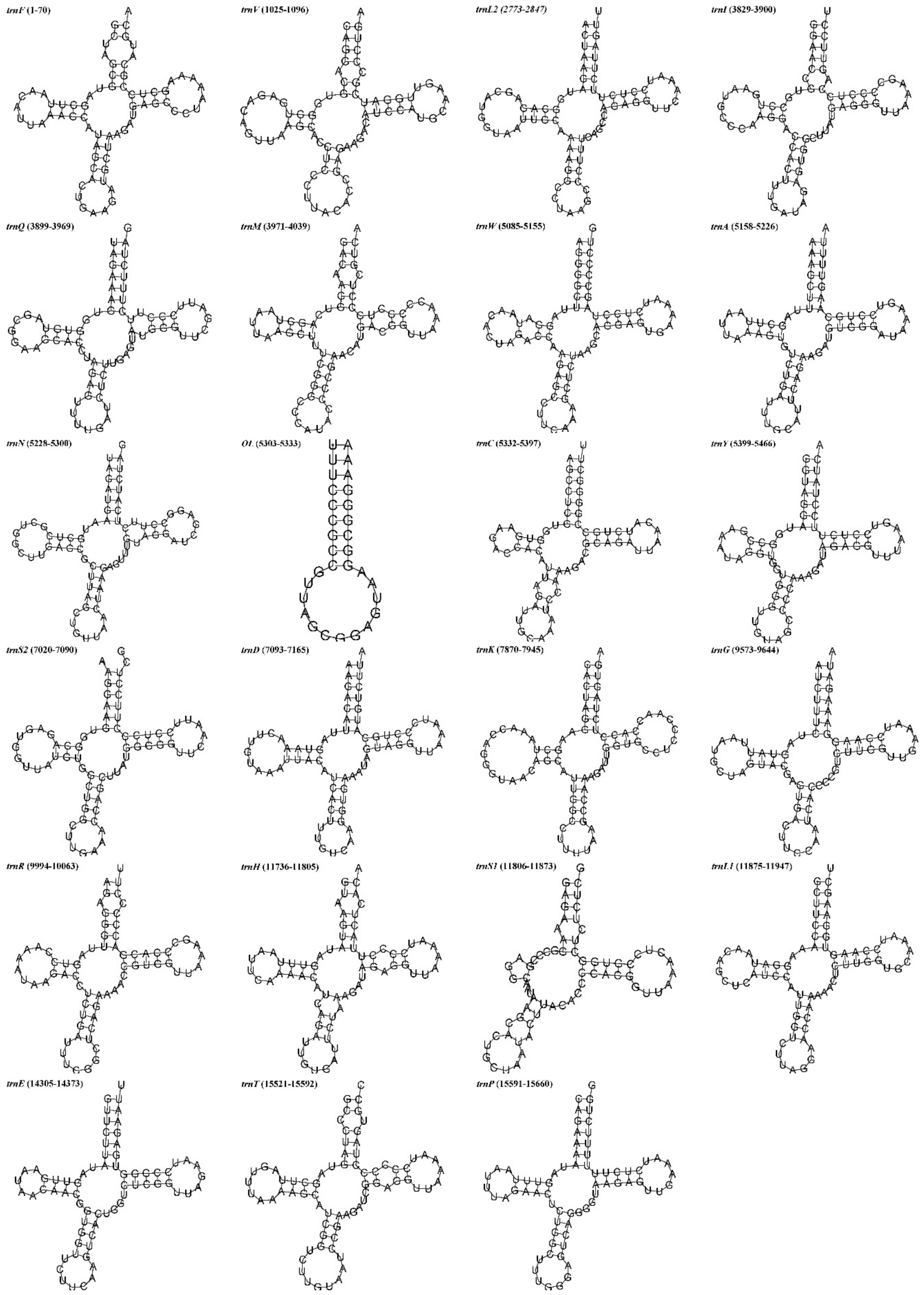

P. fangi:

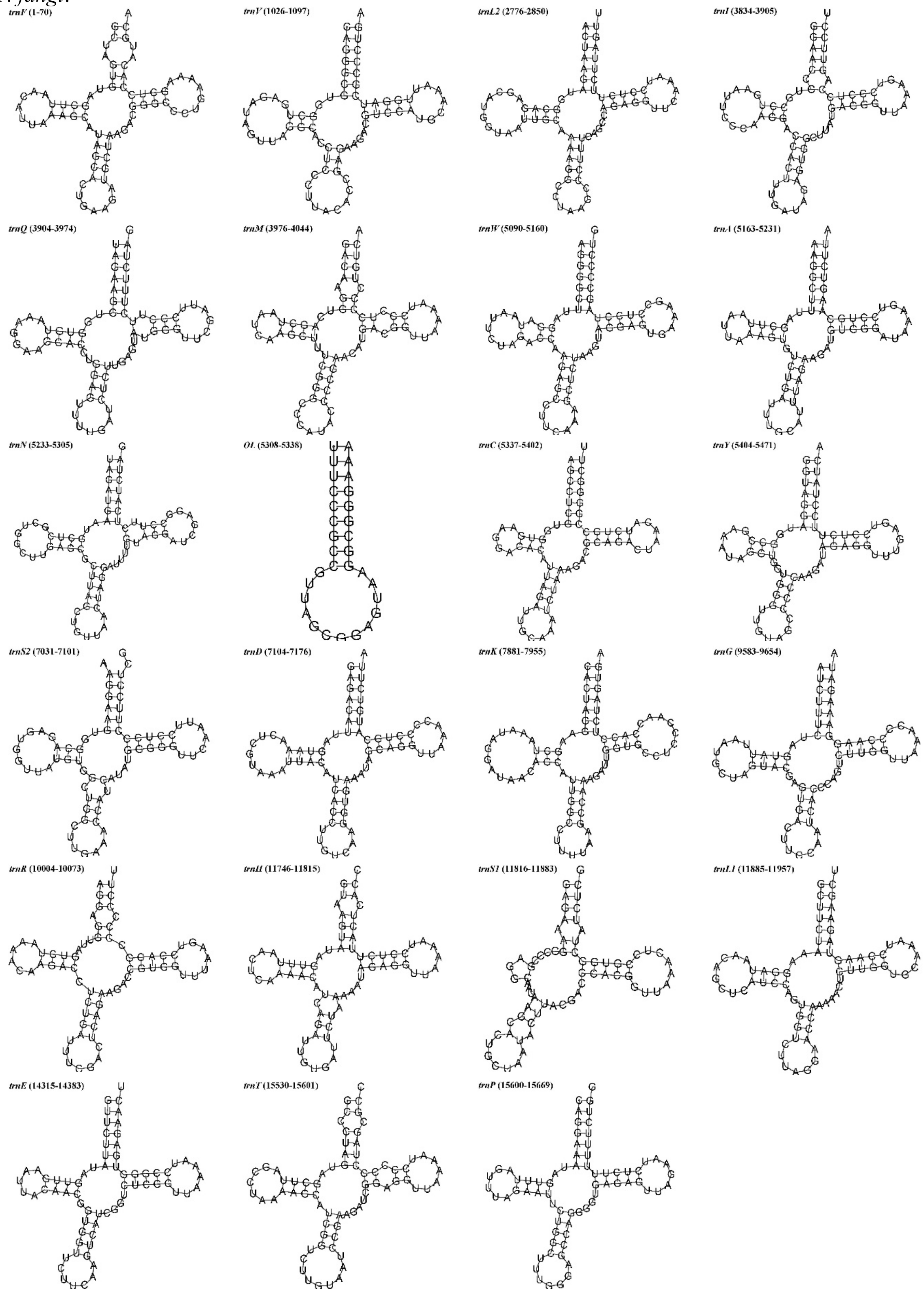
trnF (1-70)
trnV (1026-1097)
trnL2 (2776-2850)
trnI (3834-3905)
trnQ (3904-3974)
trnM (3976-4044)
trnW (5090-5160)
trnA (5163-5231)
trnN (5233-5305)
OL (5308-5338)
trnC (5337-5402)
trnY (5404-5471)
trnS2 (7031-7101)
trnD (7104-7176)
trnK (7881-7955)
trnG (9583-9654)
trnR (10004-10073)
trnH (11746-11815)
trnS1 (11816-11883)
trnL1 (11885-11957)
trnE (14315-14383)
trnT (15530-15601)
trnP (15600-15669)

P. fasciatus fasciatus:

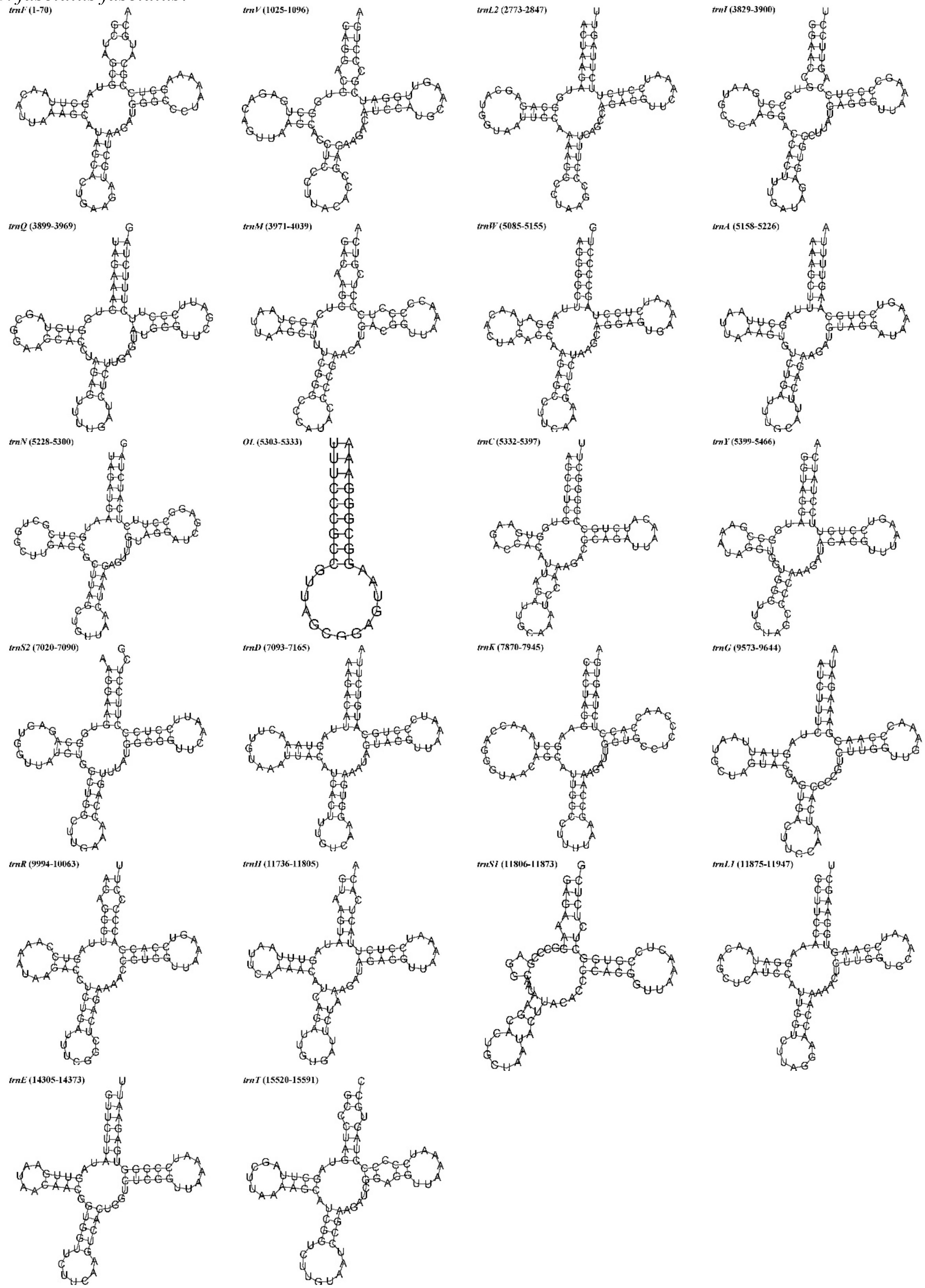

P. meihuashanensis:

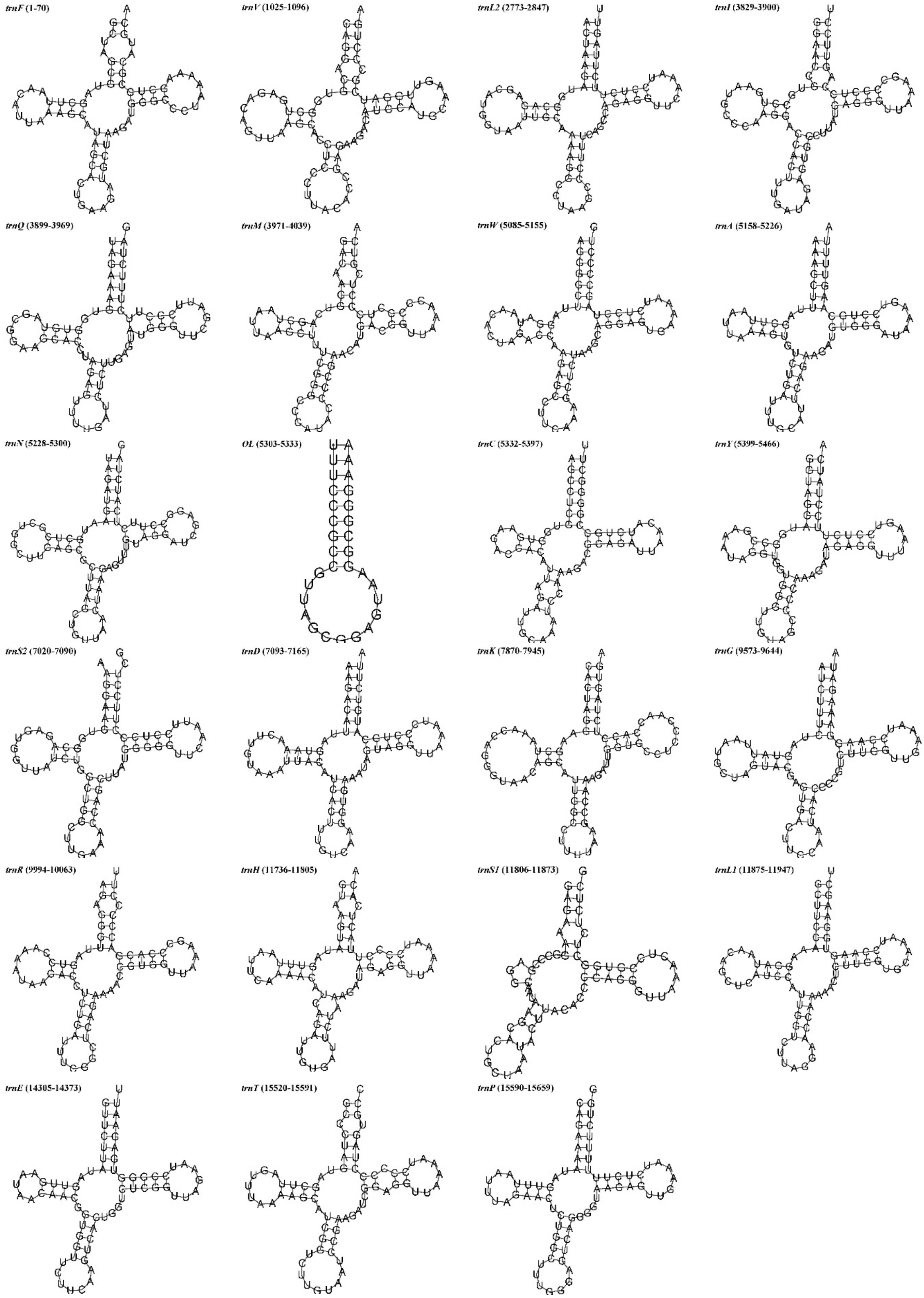

P. changtingensis changtingensis:

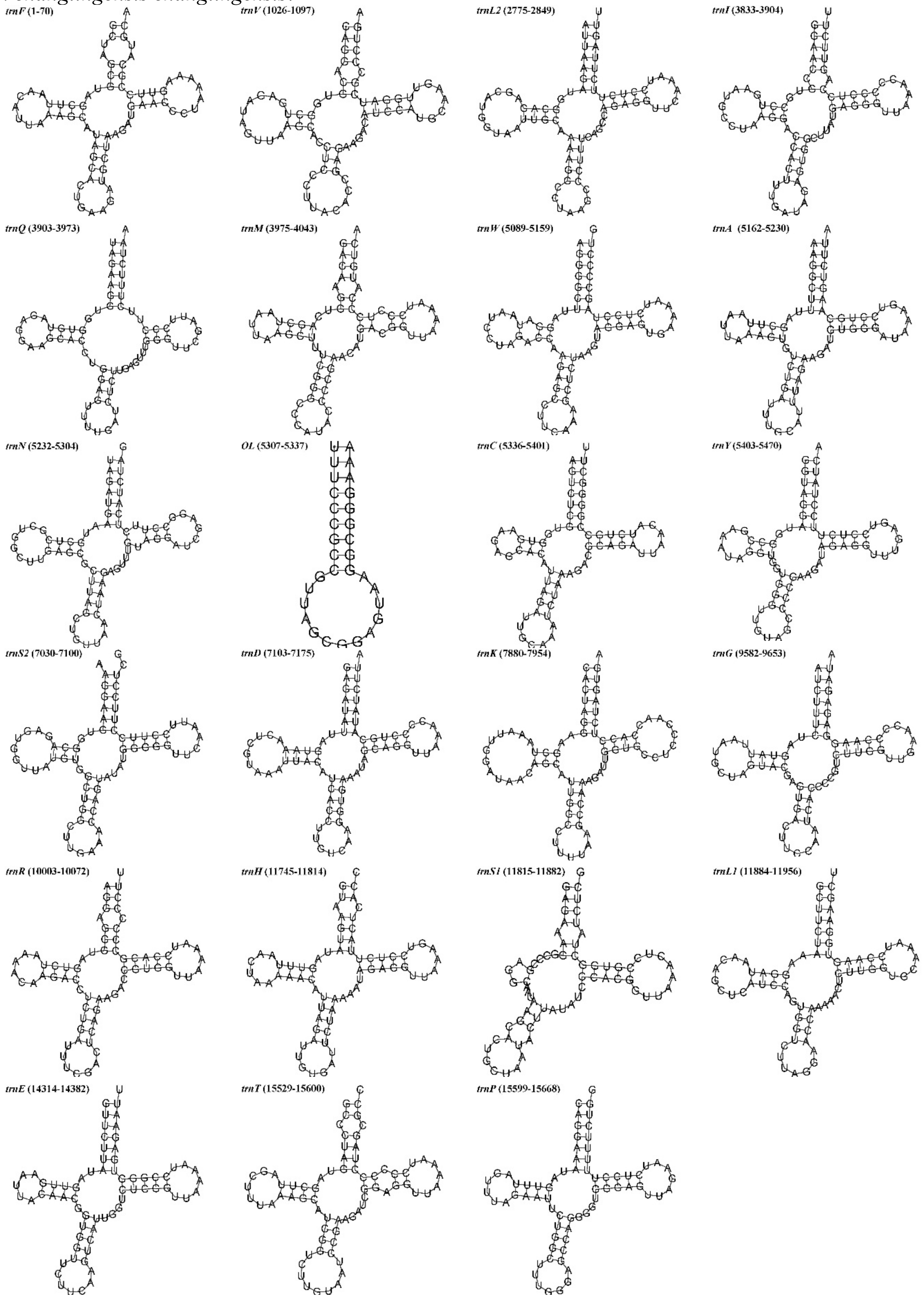

P. changtingensis tungpeiensis:

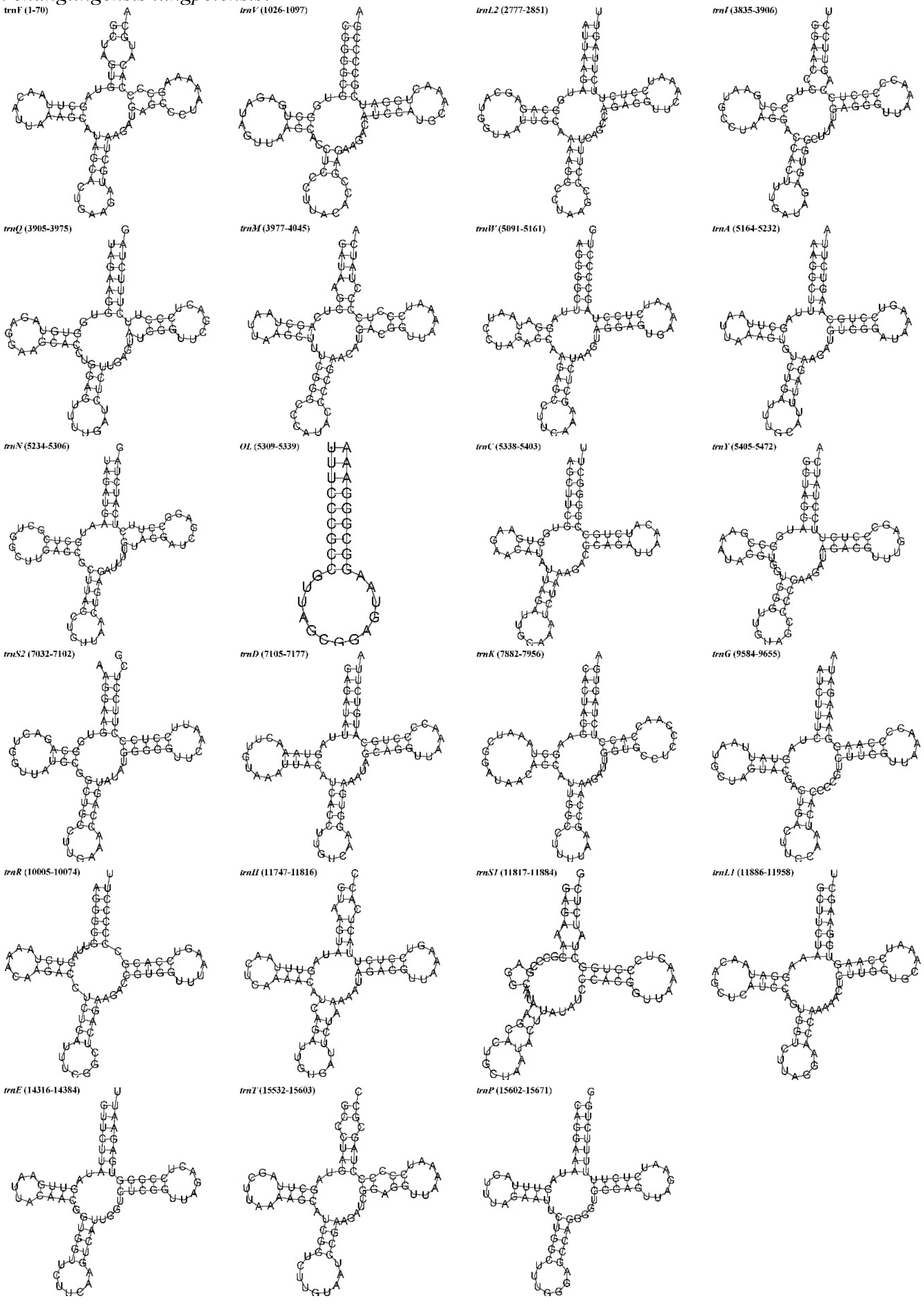

P. cheni:

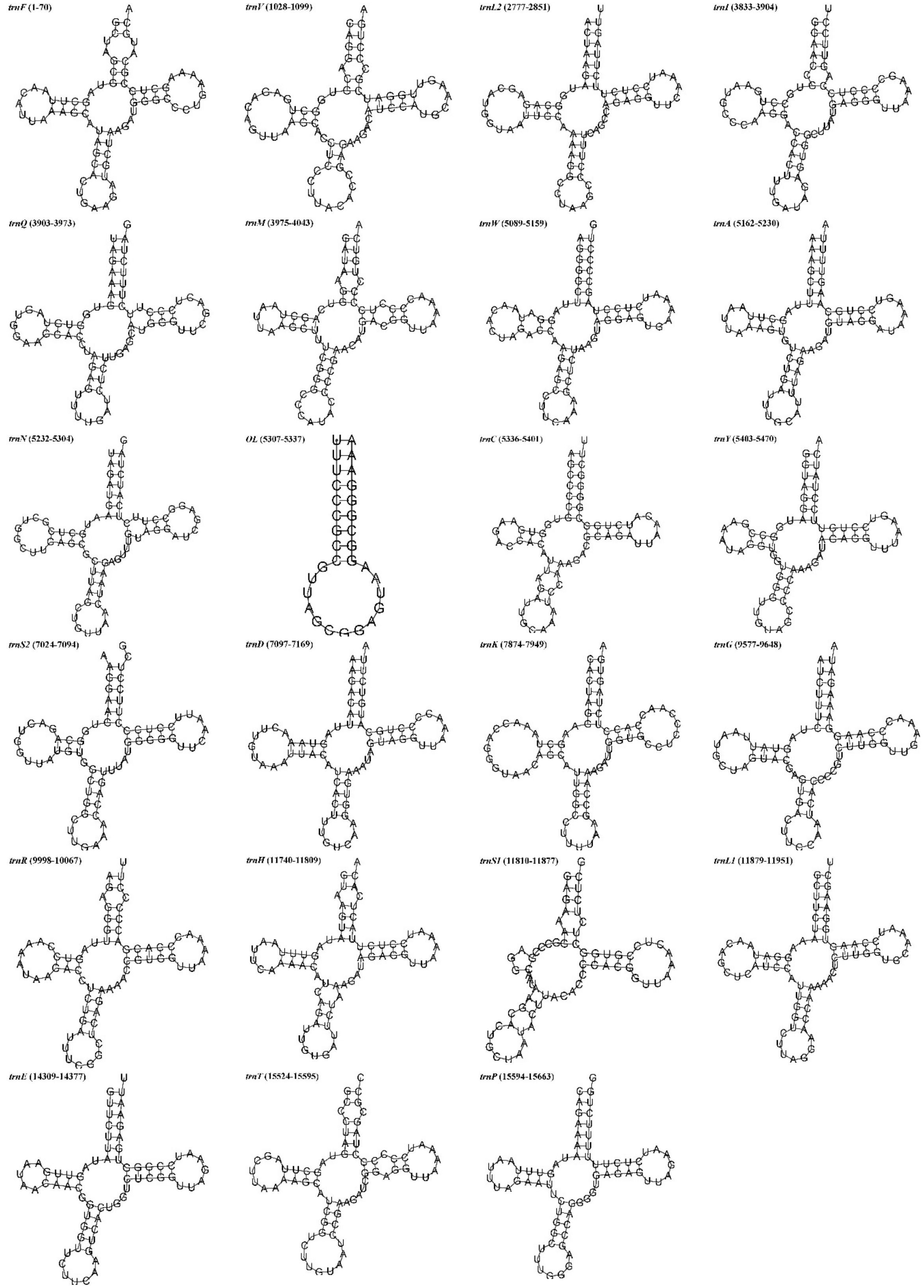

trnF (1-70)
trnV (1028-1099)
trnL2 (2777-2851)
trnI (3833-3904)
trnQ (3903-3973)
trnM (3975-4043)
trnW (5089-5159)
trnA (5162-5230)
trnN (5232-5304)
OL (5307-5337)
trnC (5336-5401)
trnY (5403-5470)
trnS2 (7024-7094)
trnD (7097-7169)
trnK (7874-7949)
trnG (9577-9648)
trnR (9998-10067)
trnH (11740-11809)
trnS1 (11810-11877)
trnL1 (11879-11951)
trnE (14309-14377)
trnT (15524-15595)
trnP (15594-15663)

P. laticeps:

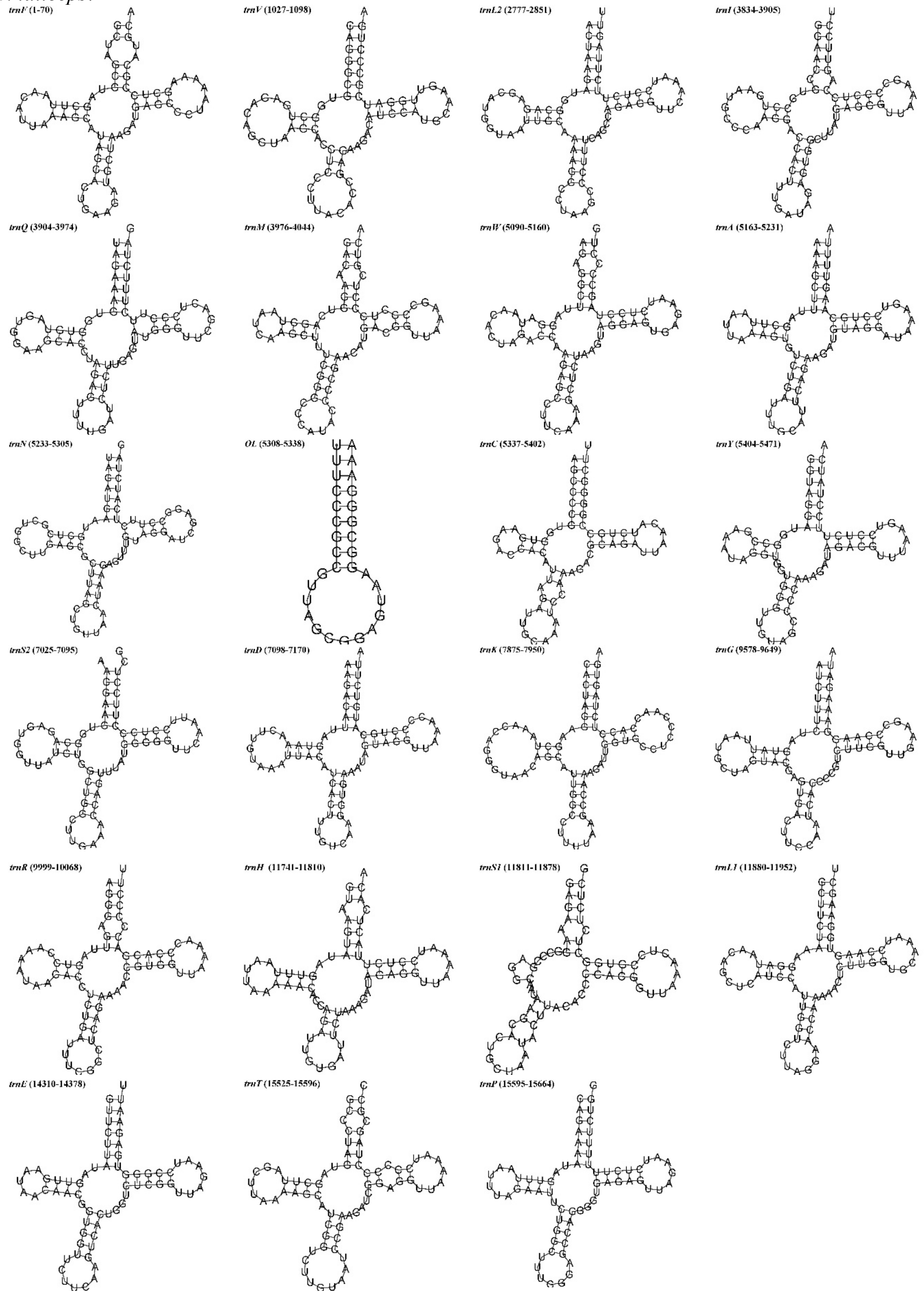

trnF (1-70)
trnV (1027-1098)
trnL2 (2777-2851)
trnI (3834-3905)
trnQ (3904-3974)
trnM (3976-4044)
trnW (5090-5160)
trnA (5163-5231)
trnN (5233-5305)
OL (5308-5338)
trnC (5337-5402)
trnY (5404-5471)
trnS2 (7025-7095)
trnD (7098-7170)
trnK (7875-7950)
trnG (9578-9649)
trnR (9999-10068)
trnH (11741-11810)
trnS1 (11811-11878)
trnL1 (11880-11952)
trnE (14310-14378)
trnT (15525-15596)
trnP (15595-15664)

P. lianjiangensis:

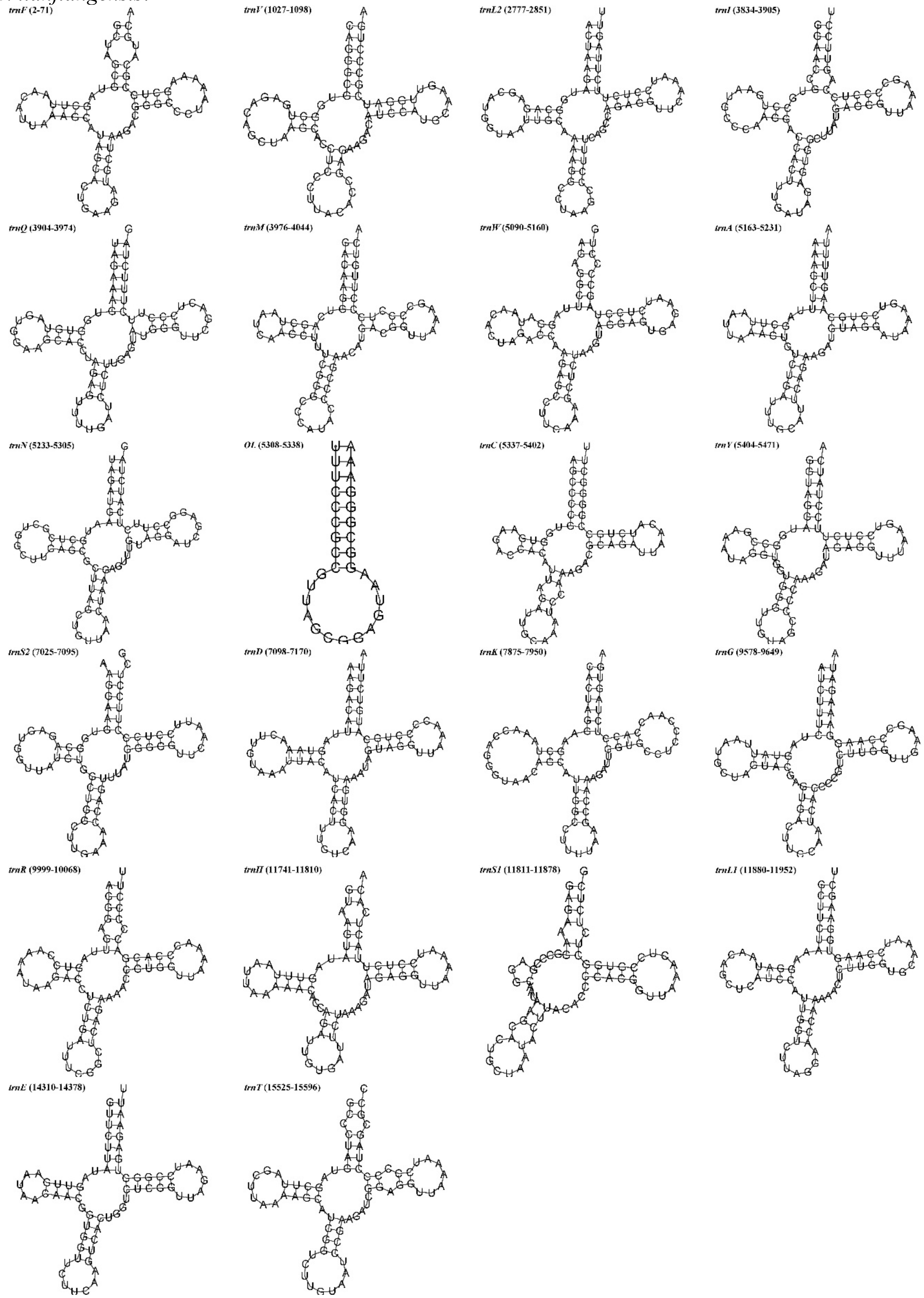

P. myersi:

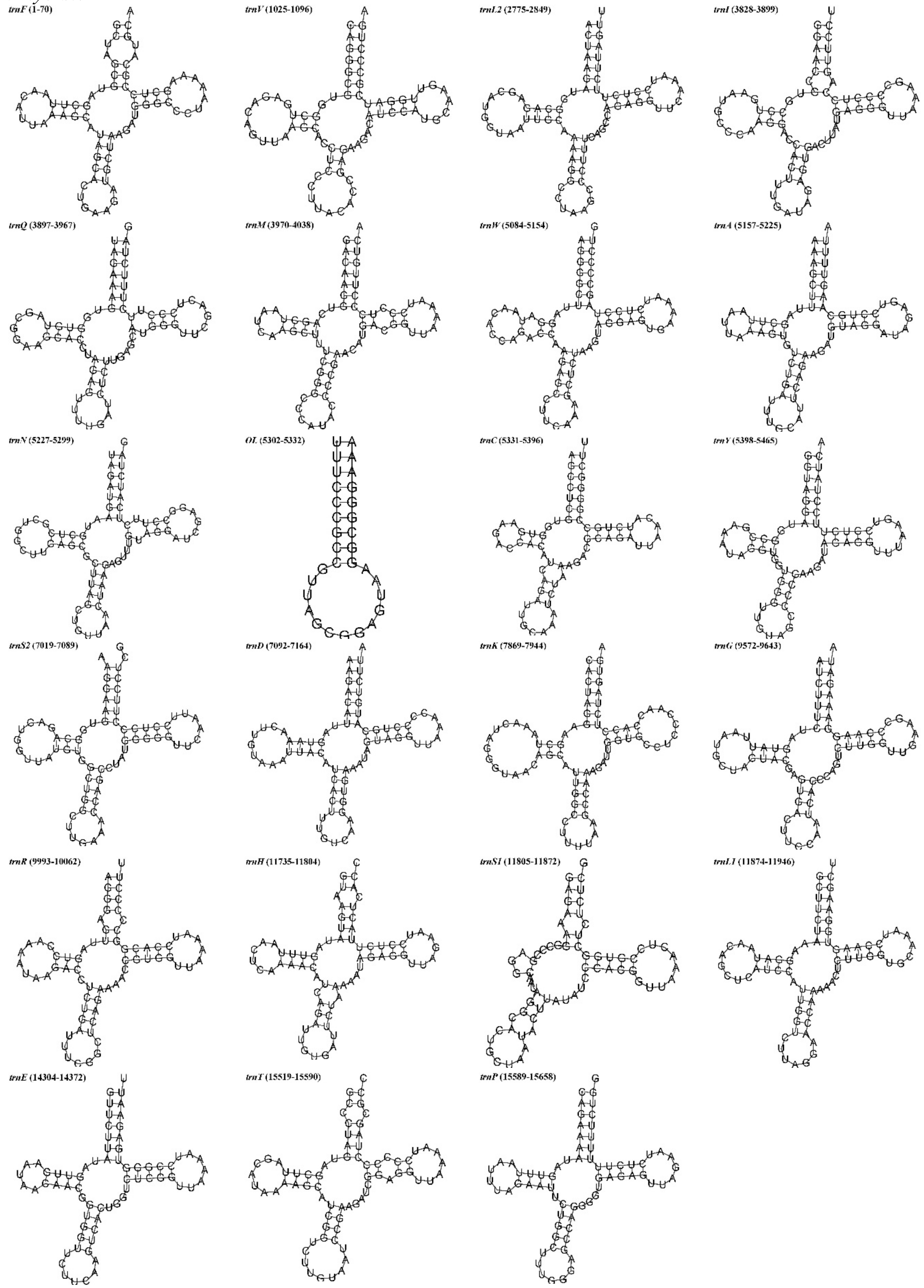

trnF (1-70)
trnV (1025-1096)
trnL2 (2775-2849)
trnI (3828-3899)
trnQ (3897-3967)
trnM (3970-4038)
trnW (5084-5154)
trnA (5157-5225)
trnN (5227-5299)
OL (5302-5332)
trnC (5331-5396)
trnY (5398-5465)
trnS2 (7019-7089)
trnD (7092-7164)
trnK (7869-7944)
trnG (9572-9643)
trnR (9993-10062)
trnH (11735-11804)
trnS1 (11805-11872)
trnL1 (11874-11946)
trnE (14304-14372)
trnT (15519-15590)
trnP (15589-15658)

P. peristictus:

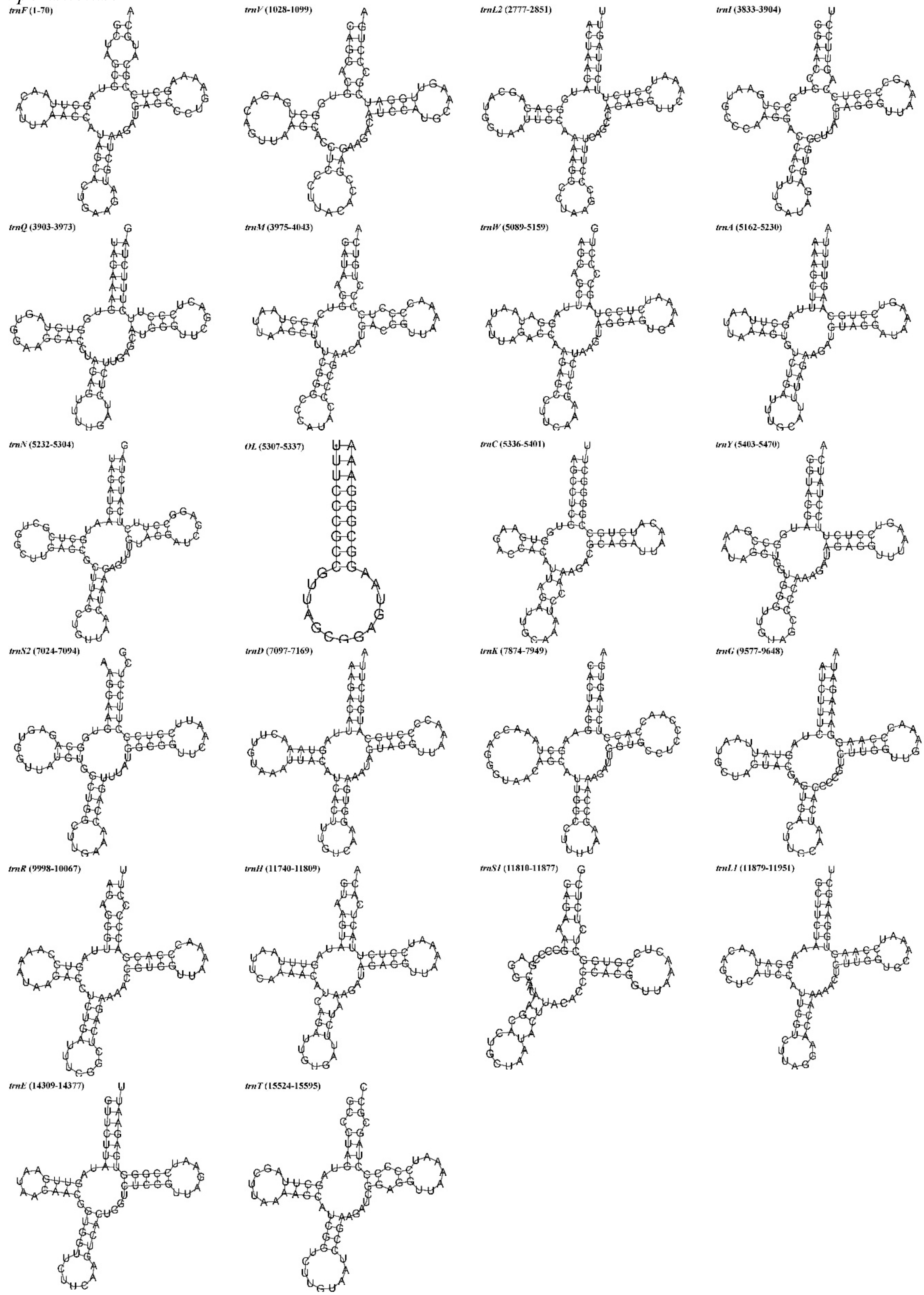

Supplement: Supplementary file 1 [file animals-14-00495-s001.zip › Figure S2.pdf]
