# Supplementary figures and images for: Comprehensive Analysis of Phylogenetic Relationship and Optimal Codons in Mitochondrial Genomes of the Genus Pseudogastromyzon
Source: Animals (Basel). 2024 Feb 2;14(3):495. doi: 10.3390/ani14030495 (PMC10854560; doi:10.3390/ani14030495)

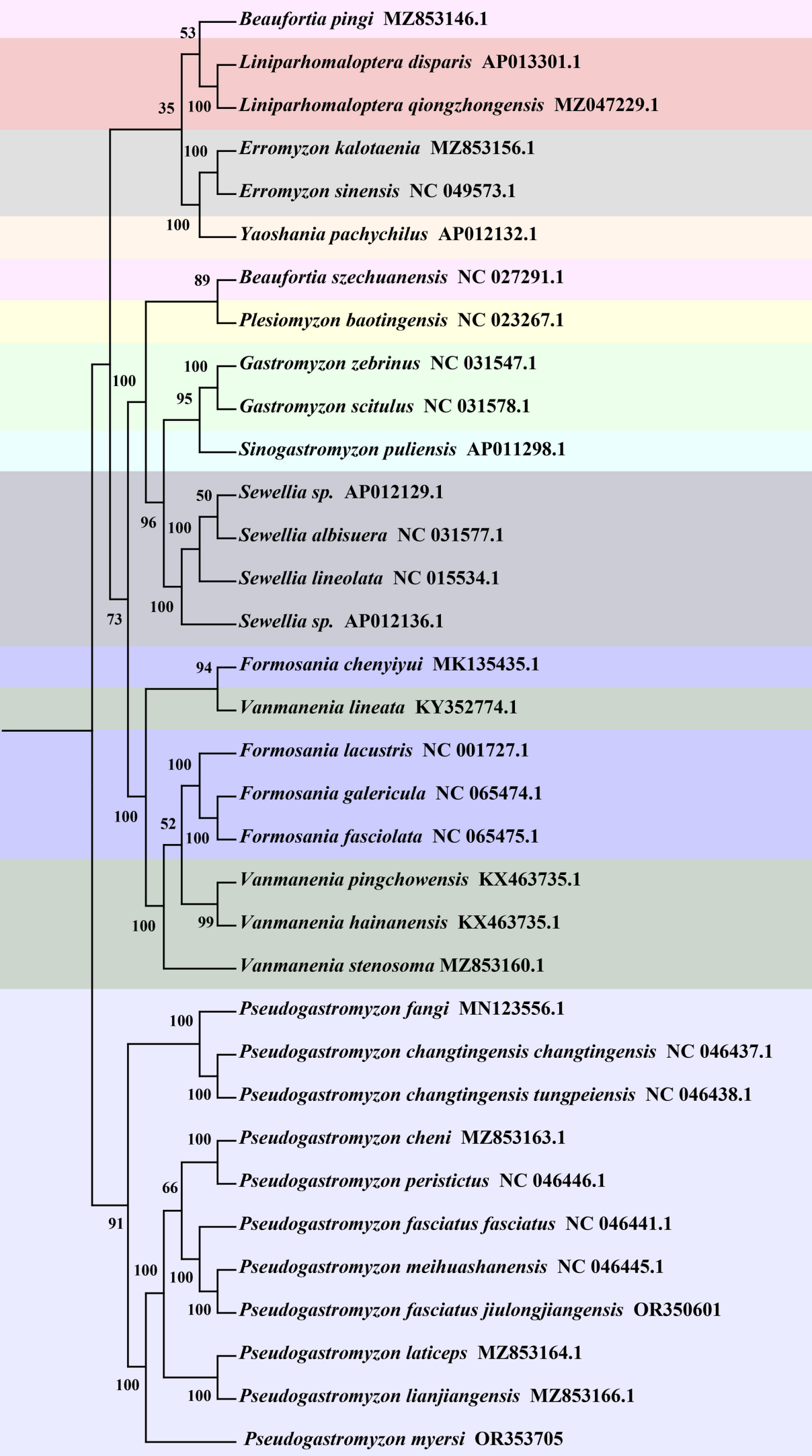

Supplement: Supplementary file 1 [file animals-14-00495-s001.zip › Figure S3.pdf]
